# ADAT2-mediated A-to-I tRNA modification promotes oncogenic translation and colorectal cancer progression and chemoresistance

**DOI:** 10.1186/s12943-026-02618-5

**Published:** 2026-03-17

**Authors:** Cillian H. Cheng, Fenfen Ji, Jianming Shen, Ying Jiao, Danyu Chen, Huan Yang, Liufang Ye, Ruizhi Tao, Qinyao Wei, Wei Kang, Jun Yu, Chi Chun Wong

**Affiliations:** 1 Department of Medicine and Therapeutics, Institute of Digestive Disease, State Key Laboratory of Digestive Disease, Li Ka Shing Institute of Health Sciences, The Chinese University of Hong Kong, Shatin, Hong Kong SAR China; 2 Department of Anatomical and Cellular Pathology, The Chinese University of Hong Kong, Shatin, Hong Kong SAR China; 3 Department of Medicine and Therapeutics, Prince of Wales Hospital, The Chinese University of Hong Kong, Shatin, NT, Hong Kong China

**Keywords:** Colorectal cancer, tRNA modification, ADAT2, Chemoresistance

## Abstract

**Background:**

Adenosine-to-Inosine (A-to-I) modification is one of the most common transfer RNA (tRNA) modifications in humans. However, the role of A-to-I tRNA modification in colorectal cancer (CRC) remains poorly understood.

**Methods:**

tRNA modification was profiled by LC-MS in paired CRC and adjacent normal tissues (*N* = 70). The clinical significance of adenosine deaminase tRNA specific 2 (ADAT2) was evaluated using in-house (*N* = 157) and TCGA cohorts (*N* = 283). The function of ADAT2 in CRC was determined in intestine-specific ADAT2 knockout mice. Mechanism of ADAT2 was assessed by integrated RNA-sequencing, tRNA-sequencing, and ribosome-sequencing analyses.

**Results:**

Among 32 tRNA nucleotide modifications, A-to-I modification is the top enriched tRNA modification in CRC tumors compared to paired adjacent normal tissues (*P* < 0.001). Consistently, A-to-I modification enzyme ADAT2 is elevated in CRC and associated with poor patient survival in independent patient cohorts. Functionally, ADAT2 overexpression promotes malignant phenotypes in CRC cells and patient-derived CRC organoids, whereas ADAT2 knockout exerts opposite effects. Intestine-specific ADAT2 knockout mice showed attenuated colorectal tumorigenesis. Integrated sequencing identified that ADAT2 boosts translation efficiency of genes highly dependent on A-to-I codons, specifically enriched in WNT/β-catenin signaling. We revealed HDAC7 as a downstream target, whereby ADAT2 promotes HDAC7 translation in an A-to-I dependent fashion. HDAC7 interacts with β-catenin, leading to its activation and nuclear translocation. For translational value, ADAT2 promotes chemoresistance in CRC, and targeting ADAT2 by VNP-encapsulated ADAT2-siRNA promoted Oxaliplatin and 5-Fluorouracil efficacy to suppress CRC growth.

**Conclusions:**

ADAT2-driven tRNA A-to-I modification promotes CRC tumorigenesis and chemoresistance via HDAC7-WNT/β-catenin axis, and is an independent prognostic factor.

**Supplementary Information:**

The online version contains supplementary material available at 10.1186/s12943-026-02618-5.

## Introduction

Colorectal cancer (CRC) is the third most prevalent cancer globally, accounting for ~ 10% of all cancer cases, and is the second leading cause of cancer-related mortality worldwide [1]. The onset and progression of cancer represent a highly intricate process, characterized by a multitude of alterations at genetic and epigenetic levels [2]. Nevertheless, the impact of epitranscriptome in contributing to tumorigenesis is just beginning to be understood. It is essential to further decipher the impact of epitranscriptome in promoting aberrant protein translation in cancer development.

Transfer RNA (tRNA) serves a vital function as an adaptor molecule, enabling translation of messenger RNA codons into their corresponding amino acids during protein synthesis [3]. tRNA modifications, recognized as the most extensively and diversely modified class of RNA across all domains of life, have received substantial and increasing attention over the past decade [4–6]. These modifications play pivotal roles in tRNA structure, folding, stability, and decoding accuracy, consequently impacting translation efficiency and overall cellular function [7–9]. In humans, dysregulation of tRNA modifications is associated with a diverse range of diseases, including cancer [10–13]. However, the potential role of tRNA modifications in colorectal tumorigenesis has yet to be comprehensively investigated.

In this study, we systematically surveyed tRNA modifications in matched CRC tumors and adjacent normal tissues, and revealed that tRNA A-to-I modification is enriched in CRC tissues. Concordantly, the A-to-I tRNA modification enzyme ADAT2 is overexpressed in CRC patients and ADAT2 high expression is associated with poor prognosis. We showed that ADAT2 promotes CRC development using CRC cell lines, CRC patient-derived organoids, and conditional, intestine-specific ADAT2 knockout (*ADAT2*^fl/fl^ *Villin-Cre*) mice. Integrated analyses of RNA-seq, tRNA-seq, and Ribo-seq unveiled that ADAT2 promotes the translation of HDAC7, which in turn, mediates activation and the nuclear translocation of β-catenin to drive colorectal tumorigenesis. Further, ADAT2 promotes WNT/β-catenin-dependent stemness in CRC cells, leading to a chemoresistant phenotype. Finally, genetic targeting of ADAT2 using intestine-specific ADAT2 knockout or vesicle-like nanoparticles (VNP)-encapsulated siRNA potentiated chemotherapy efficacy (5-Fluorouracil: 5-FU and Oxaliplatin: OXA) to suppress CRC growth, inferring ADAT2 as a potential therapeutic target in CRC.

## Materials and methods

Detailed methods are described in supplemental methods.

## Results

### A-to-I tRNA modification and its catalyzing enzyme ADAT2 are up-regulated in CRC and predicts poor survival

**Fig. 1:**
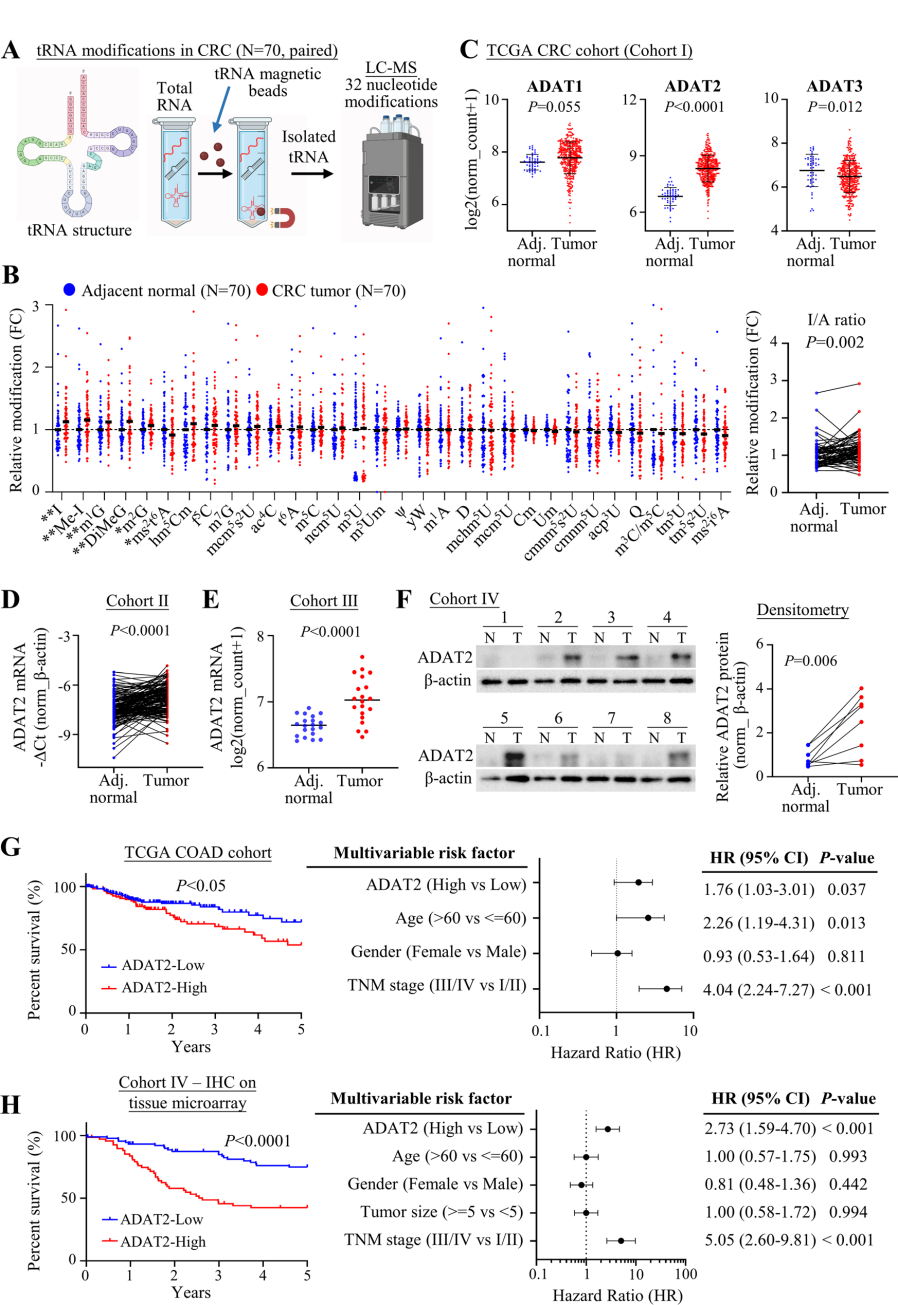
ADAT2 is overexpressed in colorectal cancer and its expression correlated with poor survival. **A** Flowchart of the process for tRNA isolation using magnetic beads and the detection of tRNA modifications by liquid chromatography-mass spectrometry (LC-MS). **B** Comparative analysis of 32 tRNA nucleotide modifications between colorectal cancer (CRC) tumors and matched adjacent normal tissues (*N* = 70 paired samples). **C** mRNA expression of ADAT1, ADAT2, and ADAT3 in CRC tumor tissues compared to adjacent normal tissues from The Cancer Genome Atlas (TCGA) cohort (Cohort I) (*N* = 380 tumors and *N* = 51 normal samples). **D** ADAT2 mRNA expression in CRC tumor tissues compared to paired adjacent normal tissues from an in-house cohort (Cohort II) (*N* = 150 paired samples). **E** ADAT2 mRNA expression in CRC tumors versus paired adjacent normal tissues from the GSE100179 cohort (Cohort III; *N* = 20 paired samples). **F** Western blot analysis of ADAT2 protein expression in paired CRC and adjacent normal tissues samples from Cohort IV. The relative protein levels of ADAT2 were normalized to β-actin. **G** Association between ADAT2 mRNA expression and overall survival in CRC patients from the TCGA cohort (Cohort I) by Kaplan Meier survival curve analysis and multivariate Cox regression with adjustments for age, gender, and TNM stage (*N* = 111 high vs. *N* = 172 low expressions). **H** Overall survival analysis based on ADAT2 protein by immunohistochemistry (IHC) on a tissue microarray cohort (TMA, Cohort IV) using Kaplan Meier survival curve analysis and multivariate Cox regression model (*N* = 68 high vs. *N* = 89 low expressions). ADAT2 mRNA expression was quantified relative to β-actin as an internal control (**D** and **E**). Association with patient survival is presented as hazard ratios (HR) and 95% confidence intervals (CI) (**G** and **H**)

To evaluate tRNA modification profiles dysregulated in CRC tumorigenesis, we conducted a comprehensive analysis of 32 modified tRNA nucleotides using liquid chromatography-mass spectrometry (LC-MS) (Fig. 1A). Among all nucleotides, inosine (I) was the most significantly overexpressed tRNA modification (*P* = 0.002), followed by methyl-inosine (me-I, *P* = 0.003) and *N*^1^-methylguanosine (m^1^G, *P* = 0.004) in CRC tumors as compared to paired adjacent normal tissues (Fig. 1B), suggesting that tRNA A-to-I modification is enriched in CRC. A-to-I deamination of tRNA occurs at 34, 37, and 57 positions. By far the most common is I_34_ [14], which increases the wobble-pairing flexibility of anticodon, enabling I_34_-tRNAs to efficiently recognize synonymous codons that end in A, C, or U. Methylated I_37_ typically involves 37 position, whilst I_57_ is only found in archaea [14]. A-to-I conversion in tRNAs is mediated by adenosine deaminase tRNA specific (ADAT) family. ADAT1 and ADAT2 respectively catalyze conversion of A_37_ and A_34_ to inosine, whereas ADAT3 is a catalytic inactive subunit of ADAT2/ADAT3 complex [15]. We thus analyzed mRNA expression of A-to-I tRNA modification regulators (ADAT1, ADAT2 and ADAT3) in The Cancer Genome Atlas (TCGA) CRC dataset (cohort I), which demonstrated that only ADAT2 was significantly upregulated in CRC (*P* < 0.0001) (Fig. 1C). To further evaluate the clinical significance of ADAT2 in CRC, we analyzed ADAT2 expression in two independent CRC cohorts. ADAT2 mRNA expression was up-regulated in 71.3% of CRC tumor tissues compared to paired adjacent normal tissues in our in-house cohort (cohort II) (*P* < 0.0001) (Fig. 1D). Additionally, ADAT2 mRNA was elevated in CRC tumor tissues compared to adjacent normal tissues in GSE100179 cohort (cohort III) (*P* < 0.0001) (Fig. 1E). Consistent with this, western blot of paired CRC and adjacent normal tissues (*P* = 0.006) (Fig. 1F) confirmed upregulation of ADAT2 at protein level.

To determine whether ADAT2 could be a prognostic factor for CRC patients, we evaluated ADAT2 expression in correlation with clinicopathological characteristics in cohorts I and IV. In cohort I, elevated ADAT2 mRNA expression was significantly correlated with poor overall survival in colon cancer patients (*P* < 0.05). Multivariate cox regression analysis was conducted to evaluate the significance of ADAT2 mRNA in prognosis along with other clinical variables including age, gender, and TNM stage. The results indicated that high ADAT2 mRNA expression was an independent prognostic factor for colon cancer (*N* = 274 with complete data; hazard ratio [HR], 1.76; 95% confidence interval [CI], 1.03–3.01; *P* = 0.037) (Fig. 1G). In the tissue microarray (TMA, cohort IV) (Figure S1A), we confirmed that high ADAT2 protein expression was associated with poor overall survival (*P* < 0.0001). After adjustment for age, gender, tumor size, and TNM stage, high ADAT2 protein retained independent prognostic significance (*N* = 157 with complete data; HR, 2.73; 95% CI, 1.59–4.70; *P* < 0.001) (Fig. 1H). Taken together, these data demonstrate that A-to-I tRNA modification and its ‘writer’, ADAT2, are both upregulated in CRC, and ADAT2 expression is a prognostic factor for poor survival.

To determine which cell type within the tumor microenvironment expresses ADAT2, we analyzed a single-cell RNA-sequencing (scRNA-seq) dataset from CRC (GSE178341). As shown in Figure S1B, ADAT2 was predominantly expressed in malignant epithelial cells, implying that ADAT2 upregulation observed in bulk tumors is primarily attributable to its overexpression in cancer cells. To ask if ADAT2 expression varies across CRC subtypes, we next stratified TCGA CRC microarray cohort (AgilentG4502A_07_3 array) according to MSI/MSS (microsatellite instability/microsatellite stability) status. However, no significant difference in ADAT2 mRNA expression was found between MSS and MSI tumors (Figure S1C), suggesting that ADAT2 upregulation is not subtype-specific.

### ADAT2 knockout inhibits CRC cell growth in vitro and in vivo

**Fig. 2:**
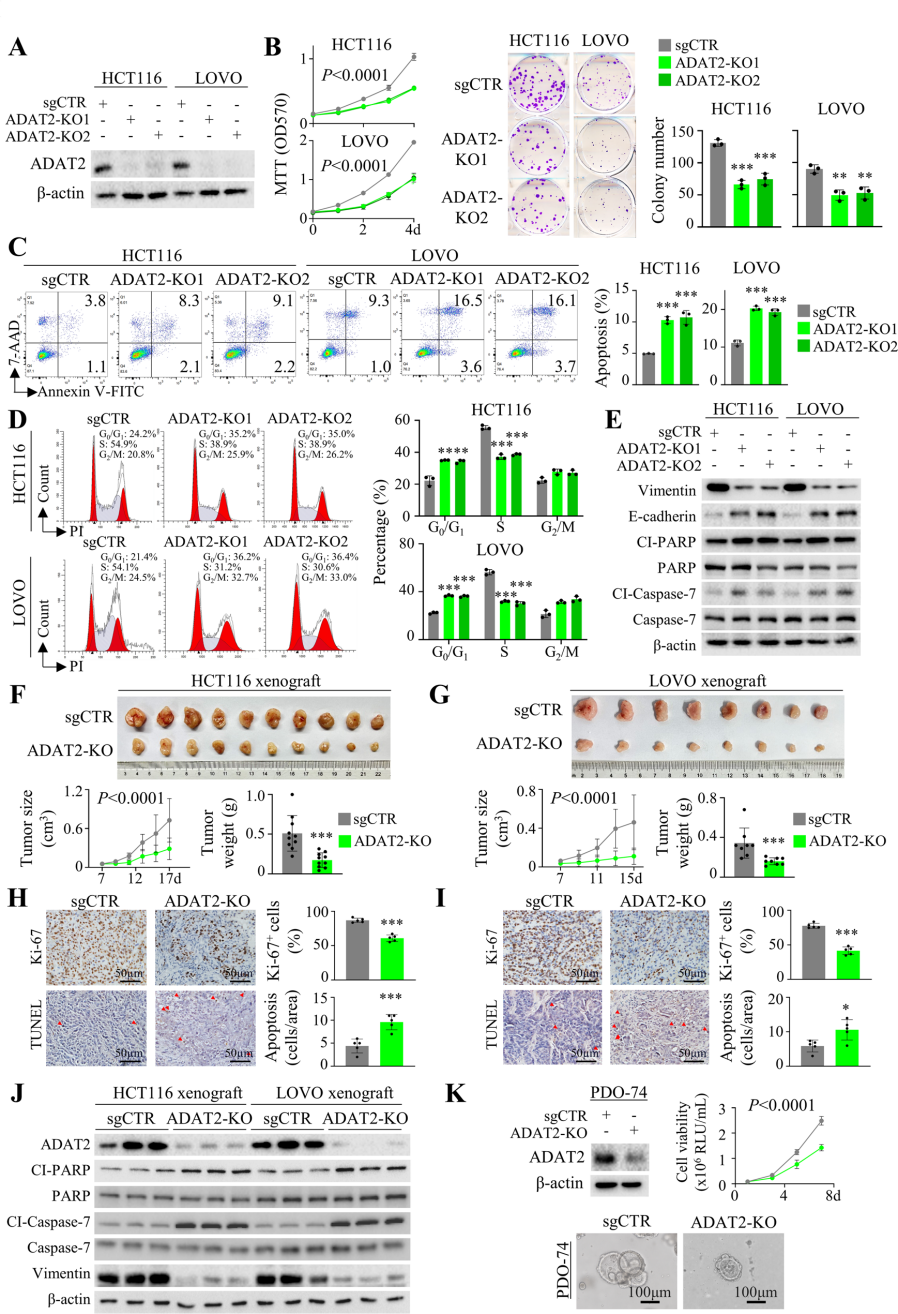
ADAT2 knockout suppresses CRC growth in vitro and in vivo. **A** ADAT2 protein expression in sgControl (sgCTR) and ADAT2-knockout HCT116 and LOVO cells. **B** Growth curves of sgCTR and ADAT2-knockout HCT116 and LOVO cells as evaluated by MTT assay (*left*) and colony formation assay (*right*) (*N* = 3). **C** ADAT2 knockout elicited an apoptosis in HCT116 and LOVO cells, as determined by Annexin V-phycoerythrin and 7-aminoactinomycin D staining and flow cytometry (*N* = 3). **D** ADAT2 knockout induced cell cycle arrest in G_0_/G_1_ phase in CRC cell lines (*N* = 3). **E** Representative western blot of apoptosis markers (cleaved caspase-7, cleaved PARP) and epithelial-mesenchymal transition (EMT) markers (E-cadherin, Vimentin) in ADAT2 knockout CRC cells. **F** HCT116 cells expressing sgADAT2 demonstrated attenuated tumor growth compared to sgCTR (*top*), both in terms of tumor volume over the assay period and tumor weight at the end point (*bottom*). Representative images are shown (*top*) (*N* = 10). **G** ADAT2 knockout in LOVO cells attenuated tumor growth compared to controls (*top*), as demonstrated by reduced tumor volume and tumor weight at the end point (*bottom*). Representative images of the tumors are shown in the upper panel (*N* = 10). **H** ADAT2 knockout HCT116 tumors exhibited reduced cell proliferation and enhanced apoptosis, as determined by Ki-67 and TUNEL staining, respectively. At least 5 fields per slide and 5 slides per group were counted at 400× magnification. **I** ADAT2 knockout LOVO tumors displayed reduced proliferation and elevated apoptosis, based on Ki-67 and TUNEL staining, respectively. At least 5 fields per slide and 5 slides per group were counted at 400× magnification. **J** Western blot analysis of HCT116 and LOVO tumors confirmed the knockout of ADAT2, the upregulation of cleaved caspase-7 and cleaved PARP, and reduced Vimentin expression. **K** Western blot confirmed ADAT2 knockout in PDO-74 patient-derived CRC organoids (*upper left*). Cell viability curves using luminescent cell viability assay (*upper right*) and representative brightfield images of PDO-74 with sgCTR or ADAT2 knockout (*bottom*) (*N* = 3). Error bars represent mean ± standard deviation. *P < 0.05; **P < 0.01; ***P < 0.001; ****P < 0.0001

To investigate the potential oncogenic role of ADAT2, we first determined its expression in CRC cell lines (Figure S2A). Next, we utilized CRISPR/Cas9 to knockout ADAT2 in HCT116 and LOVO cells with high endogenous ADAT2 expression (Fig. 2A). ADAT2 knockout suppressed the growth of CRC cells, as evidenced by cell viability and colony-formation assays (Fig. 2B). Moreover, ADAT2 knockout promoted apoptosis (Fig. 2C) and caused cell cycle arrest at G_1_/S phase (Fig. 2D). Beyond its effect on growth, ADAT2 knockout in CRC cells inhibited cell migration, matrigel invasion (Figure S2B-S2C), and wound healing (Figure S2D). Consistently, ADAT2 knockout led to increased expression of apoptosis markers, including cleaved caspase-7 and cleaved PARP, together with increased E-cadherin but reduced Vimentin expression (Fig. 2E).

To validate our results in vivo, we conducted subcutaneous xenograft studies in nude mice using HCT116 and LOVO cells. Consistently, knockout of ADAT2 significantly decreased the tumor volume and weight of HCT116 (Fig. 2F) and LOVO xenografts (Fig. 2G). Accordingly, ADAT2 knockout suppressed cell proliferation and induced apoptosis in both HCT116 (Fig. 2H) and LOVO (Fig. 2I) xenografts, as shown by Ki-67 and TUNEL staining, respectively. Western blot showed that ADAT2 knockout upregulated expression of cleaved caspase-7 and cleaved PARP, but reduced Vimentin expression in HCT116 and LOVO xenografts (Fig. 2J). Furthermore, knockout of ADAT2 in primary CRC-derived organoids (PDO-74) inhibited their growth (Fig. 2K and Figure S2E). Collectively, in vitro and in vivo studies demonstrated that ADAT2 knockout inhibits CRC growth.

### Colon-specific ADAT2 knockout attenuates colorectal tumorigenesis in mice

**Fig. 3:**
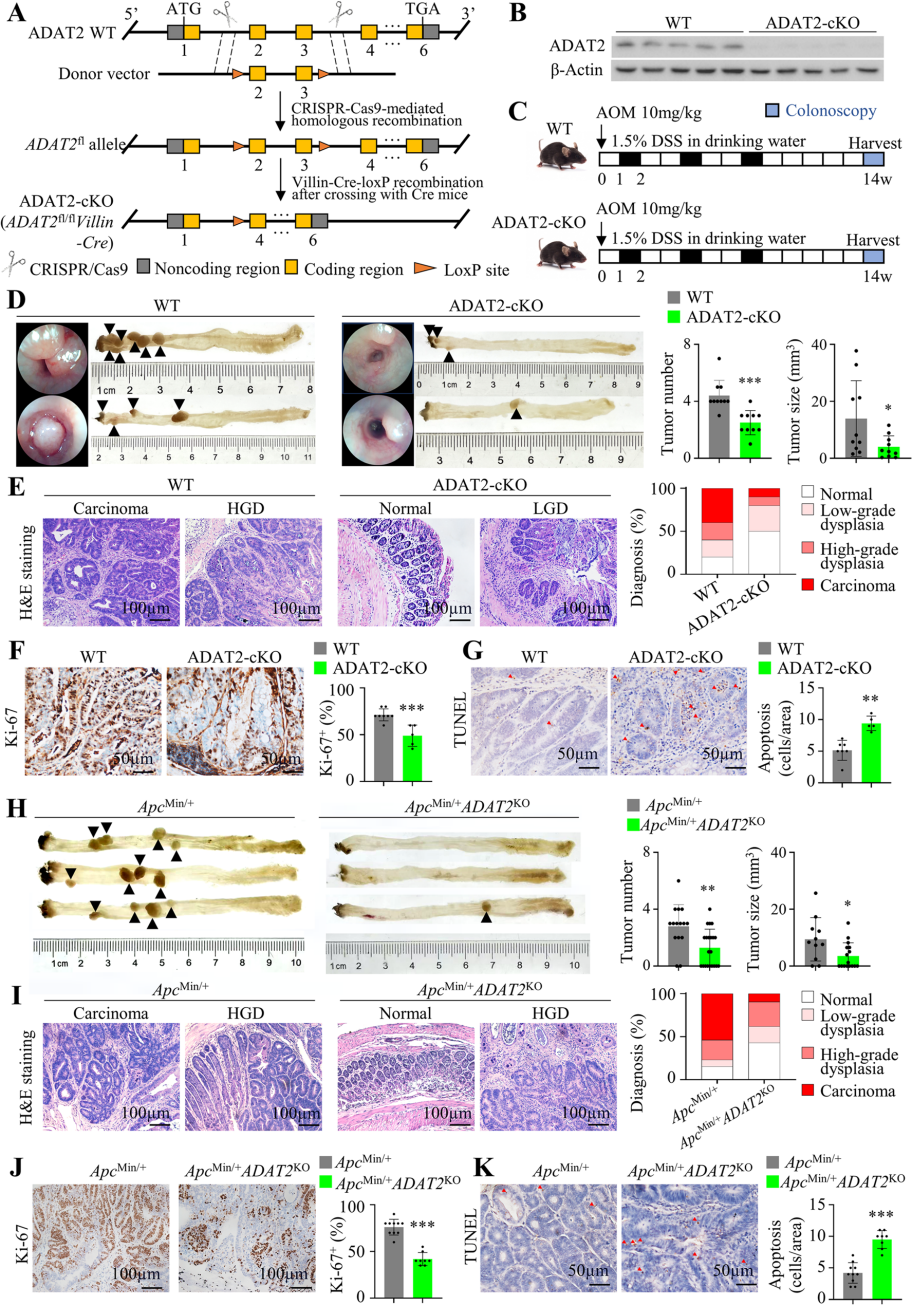
Intestine-specific ADAT2 knockout attenuates colorectal tumorigenesis in mice. **A** Construction of conditional, intestine-specific ADAT2 knockout (ADAT2-cKO) mouse model by breeding *ADAT2*^fl/fl^ mice with *Villin-Cre* transgenic mice. **B** Western blot analysis of ADAT2 expression in colon tissues from ADAT2-cKO mice and wildtype (WT) littermates (*N* = 5). **C** ADAT2-cKO mice and WT littermates (8 weeks-old, N = 10 per group) were injected azoxymethane (AOM) (10 mg/kg, intraperitoneal), followed by 3 cycles of DSS (1.5% in drinking water). Mice were sacrificed on week 14. **D** Representative images of colon from ADAT2-cKO mice and WT littermates (*left*) and measurement of tumor number and load (*right*) (*N* = 10 per group). **E** Hematoxylin and eosin (H&E) staining and histological scoring of colon from ADAT2-cKO mice and WT littermates. **F** Ki-67 immunohistochemistry (IHC) of colon tumors from ADAT2-cKO mice (*N* = 5) and WT littermates (*N* = 8). **G** TUNEL staining of colon tumors from ADAT2-cKO mice (*N* = 5) and WT littermates (*N* = 8). **H** Representative images of colon from *Apc*^Min/+^ (*N* = 15) and *Apc*^Min/+^*ADAT2*^KO^ mice (*N* = 21) (*left*), and the measurement of tumor number and load (*right*). **I** H&E staining and histological scoring of colon from *Apc*^Min/+^ and *Apc*^Min/+^*ADAT2*^KO^ mice. **J** Ki-67 IHC of colon tumors from *Apc*^Min/+^ (*N* = 10) and *Apc*^Min/+^*ADAT2*^KO^ mice (*N* = 8). **K** TUNEL assay of colon tumors from *Apc*^Min/+^ (*N* = 10) and *Apc*^Min/+^*ADAT2*^KO^ mice (*N* = 8). *P < 0.05; **P < 0.01; ***P < 0.001; ****P < 0.0001

Next, we constructed colon-specific ADAT2 conditional knockout (cKO) mice by crossing *ADAT2*^fl/fl^ mice with *Villin-Cre* mice to generate *ADAT2*^fl/fl^*Villin-Cre* mice (Fig. 3A and Figure S3A). Western blot validated knockout of ADAT2 in colon tissues (Fig. 3B), and LC-MS demonstrated that A-to-I modification was reduced (Figure S3B). Colon-specific ADAT2-cKO mice and wildtype (WT) littermates were given a single dose of carcinogen azoxymethane (AOM), together with 3 cycles of dextran sulfate sodium (DSS) to initiate CRC (Fig. 3C). Both colonoscopy and macroscopic analysis revealed that ADAT2-cKO mice had decreased tumor number (*P* < 0.001) and load (*P* < 0.05) compared to WT mice (Fig. 3D). ADAT2-cKO reduced the proportion of carcinoma and high-grade dysplasia compared to WT mice, as evidenced by H&E and histological evaluation (Fig. 3E). Concordantly, tumor tissues from ADAT2-cKO mice revealed down-regulated proliferation (*P* < 0.001, Fig. 3F) and elevated apoptosis (*P* < 0.001, Fig. 3G) compared to WT mice. Notably, untreated *ADAT2*^fl/fl^*Villin-Cre* mice have normal development and body weight (Figure S3C). Histological analysis of the colon revealed an intact crypt-villus architecture and no signs of inflammation or dysplasia (Figure S3D), suggesting that ADAT2 loss is well-tolerated in normal intestinal epithelium.

To further validate the function of ADAT2 in colorectal tumorigenesis in vivo, we crossed ADAT2-KO (*ADAT2*^+/−^) mice (Figure S3E) to *Apc*^Min/+^ mice, a genetic model for spontaneous CRC. As compared to *Apc*^Min/+^ mice, *Apc*^Min/+^*ADAT2*^KO^ mice exhibited a significantly reduced tumor number (*P* < 0.01) and load (*P* < 0.05) (Fig. 3H). *Apc*^Min/+^*ADAT2*^KO^ mice had a lower proportion of carcinoma (9.5%) compared to *Apc*^Min/+^ mice (53.8%) (Fig. 3I). IHC validated down-regulated ADAT2 protein expression (Figure S3F) and Ki-67 and TUNEL staining indicated decreased proliferation and enhanced apoptosis in tumors derived from *Apc*^Min/+^*ADAT2*^+/−^ mice (Fig. 3J-K). Together, our results showed that ADAT2 knockout in mice suppressed colorectal tumorigenesis. 

### ADAT2 overexpression promotes CRC in vitro and in vivo

**Fig. 4:**
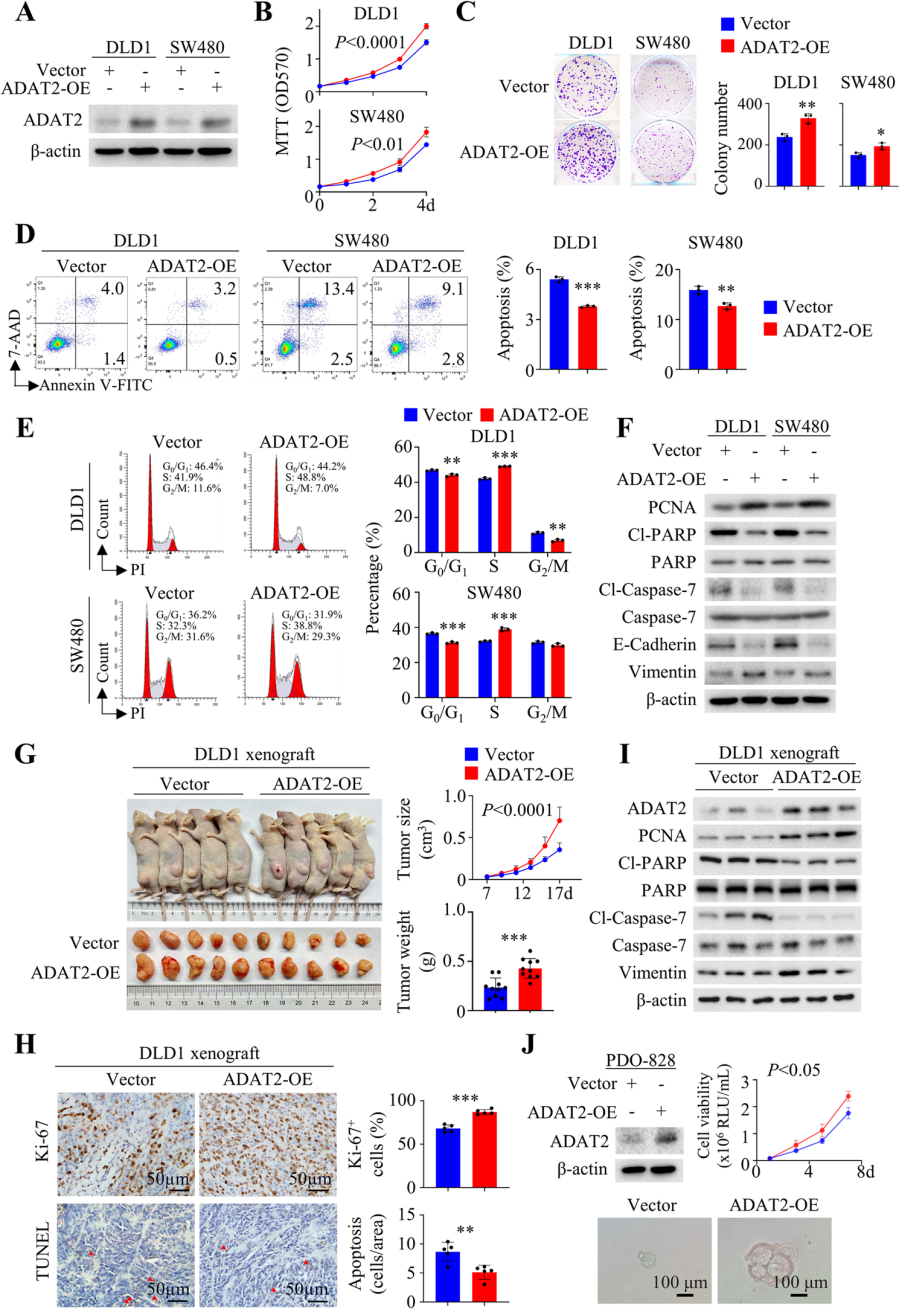
ADAT2 overexpression promotes CRC growth in vitro and in vivo. **A** ADAT2 protein expression in ADAT2-overexpressing and vector control CRC cells. **B** Growth curves of CRC cells with or without ADAT2 overexpression (*N* = 3). **C** Colony-formation assay of CRC cells with or without ADAT2 overexpression (*N* = 3). **D** Apoptosis assay of control and ADAT2-overexpressing CRC cells (*N* = 3). **E** Cell cycle analysis of control and ADAT2-overexpressing CRC cells (*N* = 3). **F** Western blot of apoptosis (cleaved caspase-7 and cleaved PARP) and EMT markers (E-cadherin and Vimentin) in ADAT2-overexpression cells. **G** Representative images (*left*), tumor growth curves (*upper right*), and tumor weight (*lower right*) of control or ADAT2-overexpressing DLD1 xenografts in nude mice (*N* = 10). **H** Ki-67 and TUNEL staining in DLD1 xenografts. **I** Western blot of cleaved caspase-7, cleaved PARP, and Vimentin in DLD1 xenografts. **J** Western blot validated the overexpression of ADAT2 in PDO-828 patient-derived CRC organoids (*upper left*). Cell viability (*upper right*) and representative brightfield images (*bottom*) of PDO-828 with vector or ADAT2 overexpression. *P < 0.05; **P < 0.01; ***P < 0.001; ****P < 0.0001

We performed gain-of-function assays by overexpressing ADAT2 in DLD1 and SW480 cells (Fig. 4A). ADAT2 overexpression increased cell growth (Fig. 4B) and colony formation (Fig. 4C), together with reduced apoptosis (Fig. 4D) and increased G_1_-S cell cycle progression (Fig. 4E). Overexpression of ADAT2 also promoted cell invasion, migration (Figure S4A-4B), and wound healing (Figure S4C-S4D). In line with these data, ADAT2 overexpression increased PCNA and Vimentin expression, but down-regulated cleaved caspase-7, cleaved PARP and E-cadherin (Fig. 4F). ADAT2 overexpression also promoted growth of DLD1 xenografts in vivo (*P* < 0.0001) (Fig. 4G), with increased cell proliferation (*P* < 0.001) and reduced apoptosis (*P* < 0.001) (Fig. 4H). Western blot confirmed upregulated PCNA and Vimentin expression, alongside the down-regulation of cleaved caspase-7 and PARP (Fig. 4I). Finally, ADAT2 overexpression increased the outgrowth of primary CRC organoids (PDO-828) (Fig. 4J). Collectively, these results suggest that ADAT2 overexpression promotes CRC progression.

### ADAT2 deaminase domain is essential for its function in promoting CRC growth

**Fig. 5:**
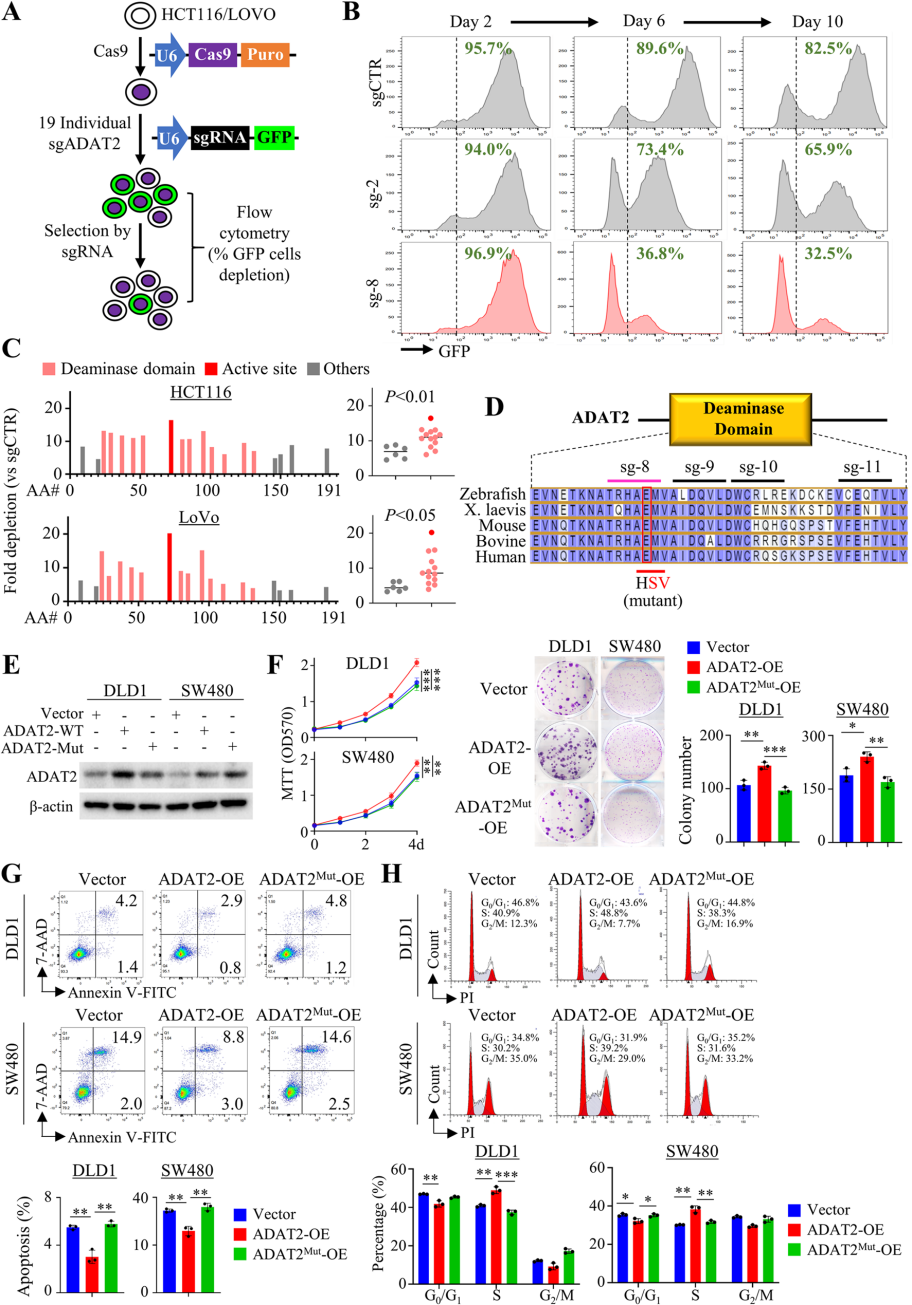
The deaminase domain of ADAT2 is essential for CRC growth. **A** Flow chart of domain-focused CRISPR screening assay. **B** CRC cells expressing ADAT2 domain-targeting sgRNA library were monitored over time based on GFP expression. Percentage of GFP^+^ cells was tracked to identify sgRNAs that impair cell viability, as indicated by a reduction in GFP^+^ fraction. **C** Systematic evaluation of 19 ADAT2 sgRNAs. The target region of each sgRNA along the length of ADAT2 protein is indicated along x-axis. **D** Evolutionary conservation of the deaminase region of ADAT2 protein. Construction of mutant ADAT2 plasmids is shown. **E** Western blot confirmed overexpression of ADAT2-WT and catalytically dead (ADAT2-Mut) ADAT2 proteins in DLD1 and SW480 cells. **F** Cell growth curves showing the effect of overexpression of ADAT2-WT and ADAT2-Mut on DLD1 and SW480 cell proliferation (*left*) and colony formation (*right*) (*N* = 3). **G** Flow cytometry analysis of Annexin V/PI staining showing the percentage of apoptotic cells in DKD1 and SW480 overexpressing ADAT2-WT and ADAT2-Mut (*N* = 3). **H** Cell cycle assay showing that the effect of overexpression of ADAT2-WT and ADAT2-Mut on cell cycle distribution in DLD1 and SW480 cells (*N* = 3). *P < 0.05; **P < 0.01; ***P < 0.001; ****P < 0.0001

To determine the functional domains of ADAT2 contributing to colorectal tumorigenesis, we utilized a domain-focused CRISPR/Cas9 strategy (Fig. 5A) [16]. Nineteen sgRNAs targeting all ADAT2 exons were evaluated, and the percentage of green fluorescent protein (GFP)^+^ cells were monitored over time to identify negative-selection phenotypes induced by sgRNAs (Fig. 5B). CRC cells expressing sgRNAs targeting sequences within the ADAT2 deaminase domain were more sensitive to negative selection (*P* < 0.01 and *P* < 0.05 in HCT116 and LOVO, respectively) compared to those targeting outside of the deaminase region (Fig. 5C). In particular, one sgRNA (sgRNA-8) was the candidate most depleted in HCT116 (16-fold depletion) and LOVO (20-fold depletion) cells (Fig. 5C). Cross-species comparison revealed the recognition sequence of sgRNA-8 lies within a conserved region containing the predicted active site residue (Fig. 5D). We thus hypothesized that pro-tumorigenic function of ADAT2 critically depends on its deaminase activity. To prove this, we generated catalytically dead ADAT2 plasmid with point mutations at the putative active sites (A72S and E73V) (Fig. 5D and Figure S5A). Immunofluorescence staining revealed that the localization of catalytic-inactive ADAT2 mutant is similar to that of wildtype ADAT2 (Figure S5B). Overexpression of wildtype ADAT2, but not catalytically dead ADAT2 (Fig. 5E), promoted DLD1 and SW480 cell growth (Fig. 5F), colony formation (Fig. 5F), cell migration, invasion (Figure S5C-S5D), and wound healing (Figure S5E-S5F). Furthermore, overexpression of ADAT2 but not its catalytically-dead mutant suppressed apoptosis (Fig. 5G) and promoted G_1_-S cell cycle progression (Fig. 5H). Taken together, these results indicate that the A-to-I tRNA deaminase activity of ADAT2 is important for its effect on CRC cells.

### Integrated tRNA-seq, RNA-seq, and Ribo-seq identifies HDAC7 as a target of ADAT2

**Fig. 6:**
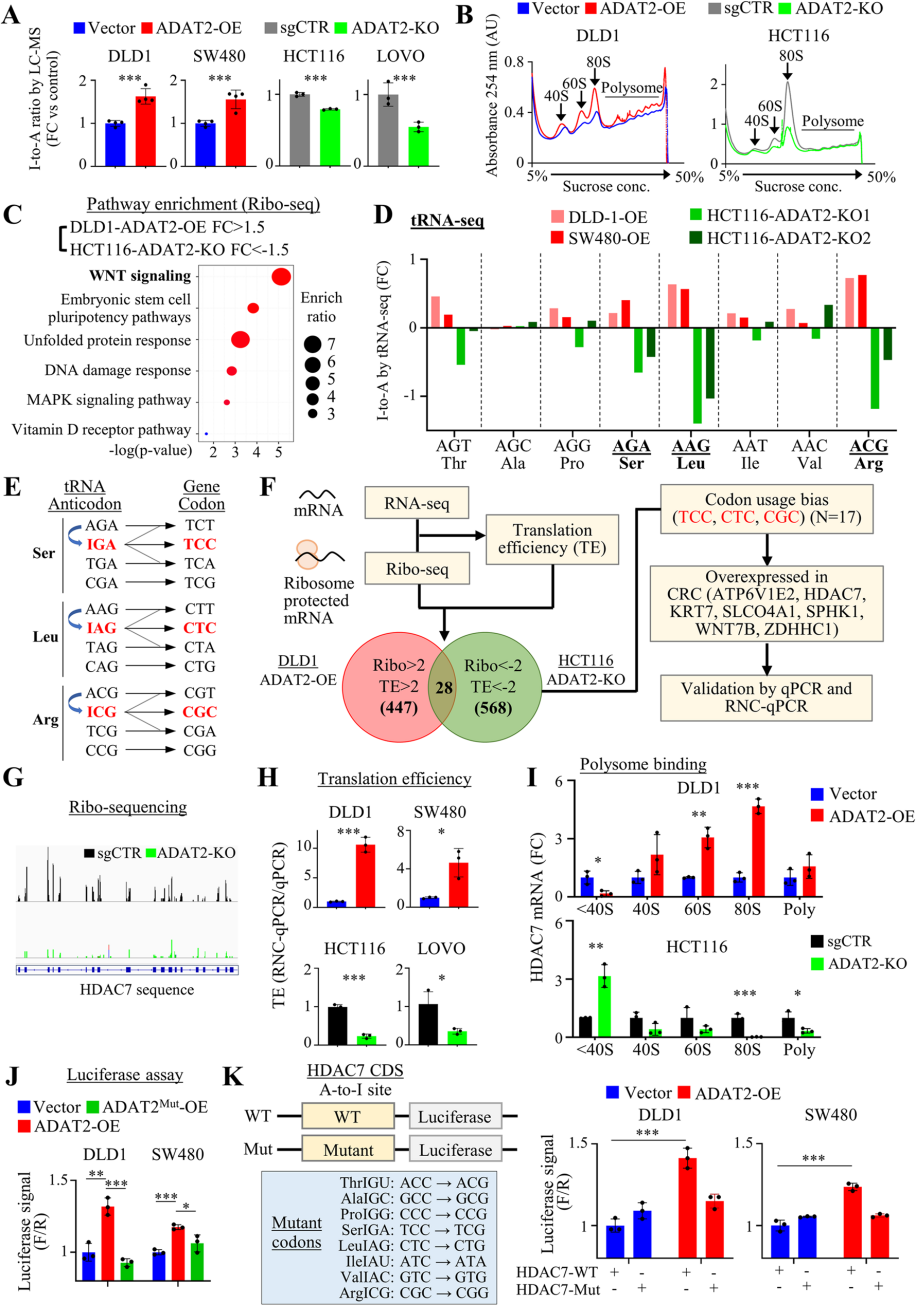
Integrative tRNA-seq, RNA-seq, and Ribo-seq analyses identify HDAC7 as a target of ADAT2. **A** Quantification of inosine-to-adenosine (I-to-A) ratio in tRNAs from DLD1 and SW480 cells overexpressing ADAT2 (*N* = 4), and HCT116 and LOVO cells with ADAT2 knockout (*N* = 3), as compared to their respective control cells. **B** Polysome profiling of DLD1 cells with ADAT2 overexpression (left) and HCT116 cells with ADAT2 knockout (right). The ribosomal subunits (40S, 60S), monosomes (80S), and polysomes were monitored at 254 nm. **C** Gene Set Enrichment Analysis (GSEA) of Ribo-seq data from ADAT2-overexpressing and ADAT2 knockout cells. The analysis revealed enrichment of genes involved in WNT signaling pathway and the regulation of pluripotency of stem cells. **D** Fold change in inosine-to-adenosine (I-to-A) modification levels in 8 tRNA anticodons targeted by ADAT2 in ADAT2-overexpressing and ADAT2 knockout cells. **E** A-to-I modification at the tRNA anticodon (e.g., AGA) allowed for wobble base pairing with corresponding mRNA codon (e.g., TCC), which is essential for the efficient decoding of these specific codons in humans. **F** Flowchart illustrating gene selection process. Candidates exhibiting altered translation efficiency (TE) and Ribo-seq abundance following ADAT2 overexpression/knockout were selected for further analysis. Subsequent filtering based on codon usage bias (TCC, CTC, CGC) and overexpression in CRC tissues narrowed down to 7 candidate genes, which were validated by qPCR and ribosome-nascent-chain complex (RNC)-qPCR. **G** Integrative Genomics Viewer (IGV) snapshots of Ribo-seq reads mapping to the exon of HDAC7 gene in control and ADAT2 knockout cells. **H** qPCR of HDAC7 mRNA and RNC-qPCR of ribosome-associated HDAC7 mRNA in CRC cells with ADAT2 overexpression or knockout. The translation efficiency (TE) was calculated as the ratio of RNC-qPCR to qPCR (*N* = 3). **I** Polysome profiling of DLD1 cells with ADAT2 overexpression and HCT116 cells with ADAT2 knockout. Fractions were collected for analysis of HDAC7 mRNA: untranslated (< 40S), translation initiation (40S, 60S, 80S), and active translation (polysomes, > 80S) (*N* = 3). **J** DLD1 and SW480 cells overexpressing ADAT2-WT or ADAT2-Mut were transfected with pcDNA3.1-HDAC7 containing the coding sequence, followed by luciferase reporter assays (*N* = 3). **K** Schematic diagram of plenti-EF1a-FH-CBH-HDAC7-WT and plenti-EF1a-FH-CBH-HDAC7-mutant luciferase reporters, the latter in which all A-to-I dependent codons were synonymously mutated to non-ADAT2-dependent codons (*left*). Luciferase activity assay showing that ADAT2 overexpression failed to enhance the translation of mutant HDAC7 reporter (*right*) (*N* = 3). *P < 0.05; **P < 0.01; ***P < 0.001; ****P < 0.0001

To study how ADAT2 modulates A-to-I tRNA modification and its consequential function on mRNA translation in CRC, we first assessed the impact of ADAT2 manipulation on A-to-I conversion in CRC cells. ADAT2 overexpression increased the I-to-A ratio (*P* < 0.001) (Fig. 6A); whereas catalytically-dead mutant ADAT2 exerted no effect (Figure S6A). Meanwhile, ADAT2 knockout down-regulated I-to-A ratio (*P* < 0.001) (Fig. 6A). Next, we performed polysome profiling to evaluate global mRNA translation activity, showing that the overexpression and depletion of ADAT2 increased and decreased 60S, 80S and polyribosome peaks in DLD1 and HCT116 cells, respectively (Fig. 6B), suggesting that ADAT2 positively regulates protein translation. To verify this, we performed puromycin incorporation assay. Our data confirmed that wildtype ADAT2, but not catalytically-dead mutant ADAT2, promoted incorporation of puromycin into nascent proteins (Figure S6B). ADAT2 knockout reduced puromycin incorporation (Figure S6C). These results indicate that ADAT2 promotes translation by promoting A-to-I modification and protein translation.

To identify the key pathways and genes whose translation are preferentially facilitated by ADAT2, we performed integrated tRNA-seq, RNA-seq, and Ribo-seq analysis. As ADAT2 is primarily involved in protein translation, we first performed gene set enrichment analysis of Ribo-seq data. Pathway enrichment analysis revealed WNT signaling and pluripotency stem cell pathways are the top pathways, whose translation were up-regulated by ADAT2 overexpression, but down-regulated upon ADAT2 knockout (Fig. 6C and Figure S7A). To decipher how manipulation of ADAT2 alters tRNA, we performed tRNA-seq. ADAT2 catalyzes A-to-I conversion of 8 anticodons in human tRNAs (tRNA^Leu^_AAG_, tRNA^Arg^_ACG_, tRNA^Ser^_AGA_, tRNA^Pro^_AGG_, tRNA^Ala^_AGC_, tRNA^Val^_AAC_, tRNA^Ile^_AAT_ and tRNA^Thr^_AGT_). Among these, ADAT2 overexpression and knockout had the most dramatic impact on A-to-I modification on tRNAs displaying the AGA (Ser), AAG (Leu), and ACG (Arg) anticodons (Fig. 6D), inferring that ADAT2 might preferentially impact the translation of genes with codon bias towards corresponding codons (TCC, CTC, and CGC) requiring A-to-I conversion to form “wobble” base pairing (Fig. 6E), which are decoded with very low efficiency in the absence of A-to-I modification in human cells [17]. We next integratively analyzed tRNA-seq, Ribo-seq and RNA-seq datasets in ADAT2 overexpression or knockout cells (Fig. 6F). First, we selected genes whose translation efficiency (TE, Ribo-seq/RNA-seq) and overall translation (Ribo-seq) were promoted by ADAT2 overexpression, whilst being decreased by ADAT2 knockout. Next, we analyzed the codon usage of 28 candidate genes to screen for genes with usage bias (TCC, CTC, and CGC) (> 1.5-fold higher than codon usage of all protein-coding genes), narrowing down to 17 candidate genes. Further, we prioritized 7 candidate genes: ATP6V1E2, HDAC7, KRT7, SLCO4A1, SPHK1, WNT7B, and ZDHHC1, that are overexpressed in CRC compared to adjacent normal samples. Many of the candidates are WNT-associated factors activated in CRC, indicating that ADAT2 promotes oncogenic translation in CRC cells.

Among these 7 candidates, IGV plot of Ribo-seq dataset revealed that HDAC7 translation in exon regions was most impacted by ADAT2 **(**Fig. 6G**)**. We validated these candidates by estimating their translation efficiency (TE), showing that the translation of HDAC7 exhibited the most consistent and pronounced change in response to ADAT2 manipulation in four CRC cell lines (HCT116 and LOVO cells with ADAT2-KO; DLD1 and SW480 cells with ADAT2-KO) (Fig. 6H and S7B). To further determine ADAT2-mediated HDAC7 translation, we performed polysome profiling and measured the abundance of HDAC7 mRNA in untranslated (< 40S), initiation (40S/60S/80S), and polysomes (> 80S) (Fig. 6I). In ADAT2-overexpressing DLD1 cells, HDAC7 mRNA was reduced in < 40S fraction but enriched in 60S/80S fractions, inferring enhanced translation initiation, whereas an opposite trend was found in HCT116 cells with ADAT2 knockout (Fig. 6I), supporting that ADAT2 promotes HDAC7 translation.

We next constructed luciferase reporters for measuring HDAC7 translation by linking its coding sequence to pcDNA3.1-luc vector (Fig. 6J). Indeed, overexpression of ADAT2 increased luciferase activities of HDAC7 reporter in CRC cells, whereas catalytically-dead mutant ADAT2 had no equivalent effects (Fig. 6J). To prove that ADAT2-mediated translation depends on HDAC7 codon bias necessitating A-to-I tRNA modifications, we generated a mutant HDAC7 luciferase vector, in which all the 8 A-to-I dependent codons were replaced with synonymous codons whose translation does not require A-to-I modification (Fig. 6K). The expression of wildtype and mutant reporter constructs were comparable at mRNA level (Figure S7C). Consistent with previous results, ADAT2 overexpression elevated wildtype HDAC7 luciferase reporter activity. However, ADAT2 failed to increase mutant HDAC7 luciferase reporter activity. Collectively, ADAT2 mediates A-to-I modification dependent translation in CRC cells, with HDAC7 as a key target.

### ADAT2 activates HDAC7-β-catenin signaling

**Fig. 7:**
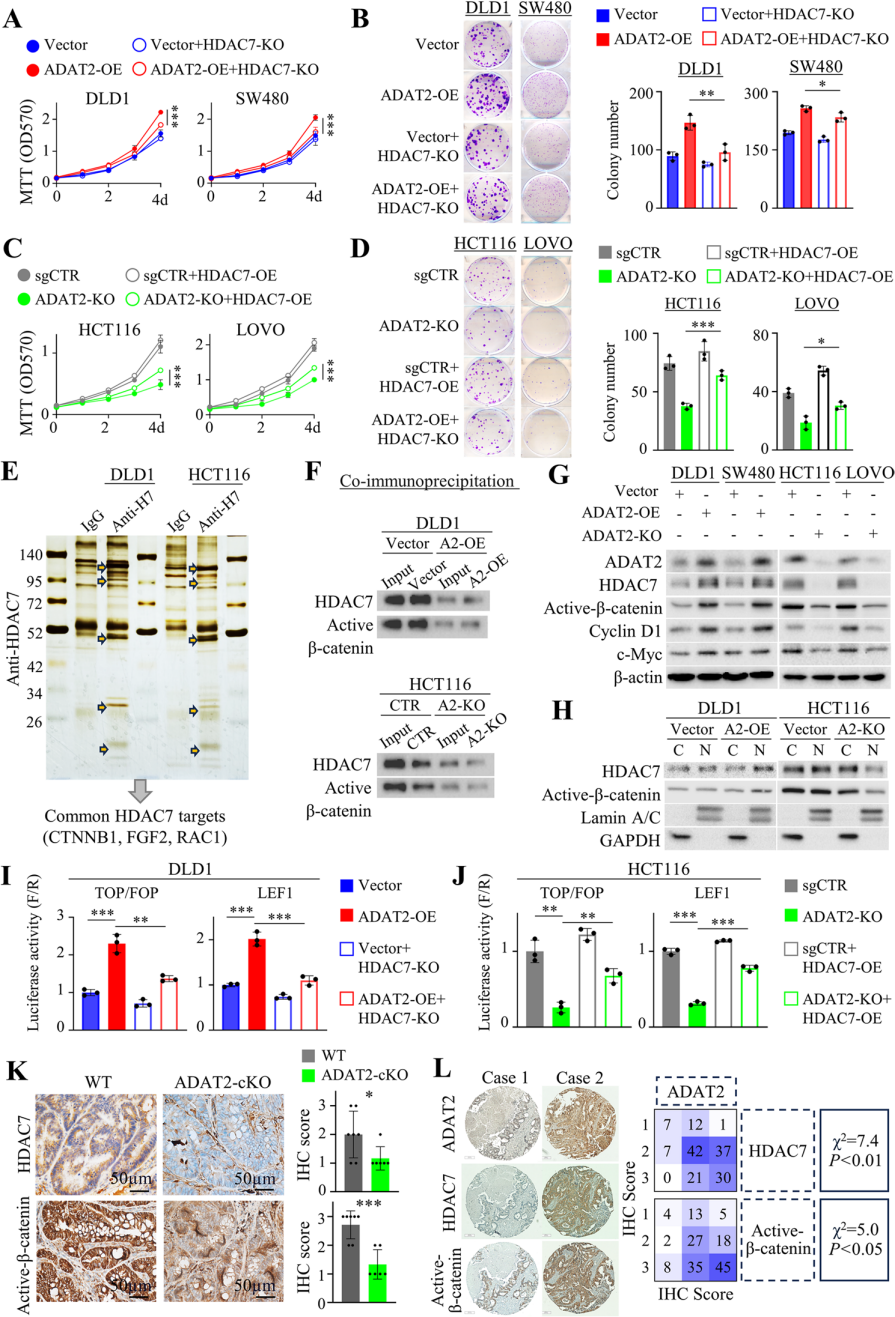
ADAT2 activates HDAC7-β-catenin signaling axis in CRC. **A** Cell growth curves of ADAT2-overexpressing DLD1 and SW480 cells, with or without HDAC7 knockout (*N* = 3). **B** Colony formation of ADAT2-overexpressing DLD1 and SW480 cells, with or without HDAC7 knockout (*N* = 3). **C** Cell growth curves of ADAT2 knockout HCT116 and LOVO cells, with or without HDAC7 overexpression (*N* = 3). **D** Colony formation of ADAT2 knockout HCT116 and LOVO cells, with or without HDAC7 overexpression (*N* = 3). **E** HDAC7 coimmunoprecipitation and mass spectrometry for identification of common interacting proteins in DLD1 and HCT116 cells. β-Catenin was identified as a top enriched protein candidate. **F** Co-immunoprecipitation assays showing the protein interaction between HDAC7 and β-catenin in CRC cells with ADAT2 overexpression or knockout. **G** Western blot of HDAC7, active β-catenin, Cyclin D1, and c-Myc proteins in CRC cells with ADAT2 overexpression or knockout. **H** Western blot of cytoplasmic (C) and nuclear (N) HDAC7 and active β-catenin proteins in CRC cells with ADAT2 overexpression or knockout. **I** Luciferase reporter assays (TOPflash/FOPflash and LEF-1) of WNT/β-catenin pathway activation in ADAT2-overexpressing DLD1 cells with or without HDAC7 knockout (*N* = 3). **J** Luciferase reporter assays (TOPflash/FOPflash and LEF-1) of WNT/β-catenin pathway status in ADAT2 knockout HCT116 cells with or without HDAC7 overexpression (*N* = 3). **K** HDAC7 and active β-catenin IHC in colon tumors from ADAT2-cKO mice (*N* = 6) and wildtype littermates (*N* = 7). **L** Representative images of ADAT2, HDAC7, and active β-catenin staining in CRC tissue microarrays (*N* = 157, Cohort IV) (*left*), Pearson correlation analysis of ADAT2, HDAC7, and active β-catenin protein expression (*right*). *P < 0.05; **P < 0.01; ***P < 0.001; ****P < 0.0001

To assess whether HDAC7 functions downstream of ADAT2, we performed HDAC7 gain-of-function and loss-of-function assays. In ADAT2-overexpressing DLD1 and SW480 cells, HDAC7 knockout abolished ADAT2-induced cell viability (Fig. 7A), colony formation (Fig. 7B), cell migration, and invasion (Figure S8A-S8B). On the other hand, ectopic overexpression of HDAC7 in ADAT2-knockout HCT116 and LOVO cells rescued cell growth (Fig. 7C), colony formation (Fig. 7D), cell migration, and invasion (Figure S8C-S8D). These results indicate that HDAC7 is a downstream factor involved in ADAT2-driven CRC.

To determine how HDAC7 functions to promote CRC, we determined the interactome of HDAC7 by employing co-immunoprecipitation (co-IP) assay with anti-HDAC7, followed by LC-MS/MS analysis of enriched protein bands. The overlap of protein candidates from DLD1 and HCT116 cells revealed several common protein candidates, of which β-catenin is the top enriched protein (Fig. 7E). co-IP demonstrated that overexpression of ADAT2 increased the interplay between HDAC7 and β-catenin, whereas ADAT2 knockout caused reduced interaction between the two proteins (Fig. 7F). As WNT/β-catenin pathway is often activated in CRC, we evaluated if ADAT2-mediated HDAC7 up-regulation promotes WNT/β-catenin signaling. Western blot revealed that ADAT2 overexpression in CRC cells promoted WNT/β-catenin signaling, as evidenced by the increased expression of active β-catenin, Cyclin D1, and c-Myc, whereas ADAT2 knockout decreased expression of these proteins (Fig. 7G). Consistently, nuclear active β-catenin was up-regulated by ADAT2 overexpression, while being down-regulated by ADAT2 knockout (Fig. 7H). TOPflash and LEF-1 luciferase assays validated increased WNT/β-catenin-mediated transcriptional activity in ADAT2 overexpressing CRC cells (Fig. 7I), with ADAT2 knockout exerting an opposite effect (Fig. 7J). Further, HDAC7 knockout abolished the effect of ADAT2 on TOPflash and LEF-1 luciferase activities (Fig. 7I). Conversely, the overexpression of HDAC7 in ADAT2-knockout CRC cells partially restored TOPflash and LEF1 luciferase activities (Fig. 7J), suggesting that HDAC7 mediates the positive effect of ADAT2 on WNT/β-catenin signaling. Activation of WNT/β-catenin by ADAT2 was further evidenced by Ribo-seq dataset showing that translation of multiple WNT targets were modulated by ADAT2 overexpression/knockout including FZD9, WNT7B, WNT10A, DVL1, and DVL3 (Figure S7A).

Next, we sought to validate ADAT2-HDAC7-WNT/β-catenin pathway in vivo. Compared to wildtype mice, ADAT2-cKO tumors demonstrated significantly decreased expression of HDAC7 (*P* < 0.05) and active β-catenin (*P* < 0.001) (Fig. 7K). In the TMA CRC cohort, we observed positive correlations between ADAT2 and HDAC7 (χ^2^ = 7.4; *P* < 0.01) or active β-catenin (χ^2^ = 5.0; *P* < 0.05) protein expression by IHC (Fig. 7L). Collectively, ADAT2 promotes WNT/β-catenin pathway through promoting HDAC7 translation. 

### Targeting ADAT2 sensitizes CRC to chemotherapy in vitro and in vivo

**Fig. 8:**
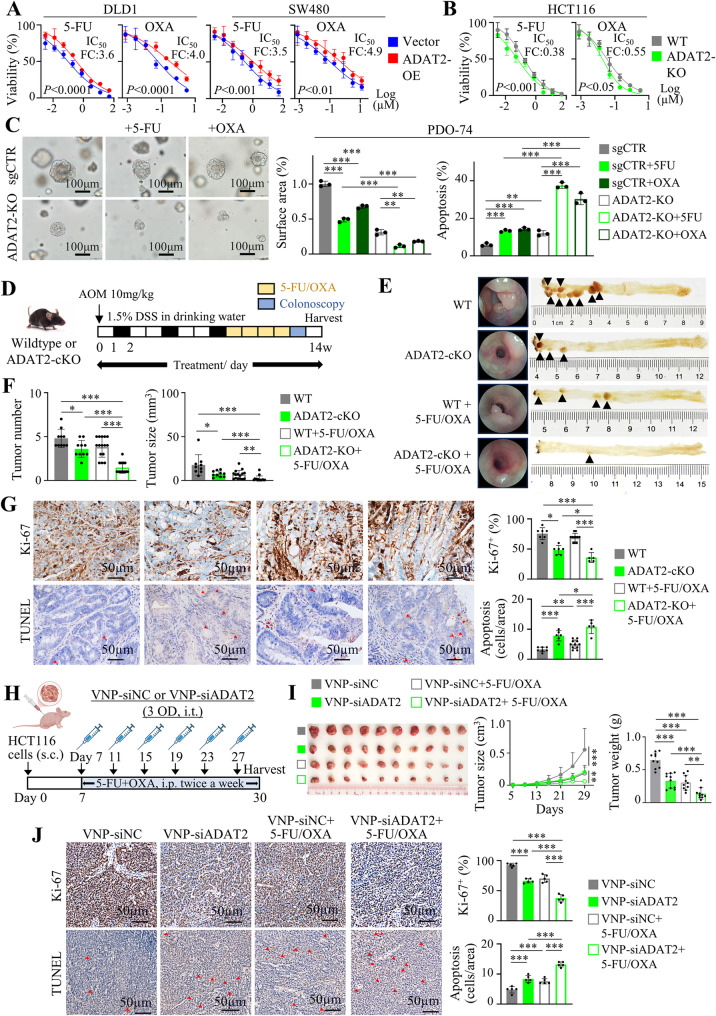
Therapeutic synergy of targeting ADAT2 plus chemotherapy in CRC. **A** 72 h-IC50 values for 5-FU and OXA in DLD1 and SW480 cells with vector control or ADAT2-overexpression. **B** 72 h-IC50 values for 5-FU and OXA HCT116 cells with sgCTR or ADAT2 knockout. **C** Effect of 5-FU and OXA on PDO-74 organoids growth and (*left*) and apoptosis (*right*) with sgCTR or ADAT2 knockout (*N* = 3). **D** Intestine-specific ADAT2 knockout mice were subjected to AOM/DSS-induced CRC, followed by treatment with chemotherapy (5-FU + OXA). **E** Representative images of colon from different treatment groups. **F** Quantification of tumor number and size in each group (*N* = 10 for untreated groups and *N* = 15 for chemotherapy-treated groups). **G** Representative Ki-67 (*top*) and TUNEL (*bottom*) staining images for AOM/DSS-induced CRC model at the endpoint. **H** Schematic diagram of HCT116 xenograft model treated with nanoparticles (VNP-siNC or VNP-siADAT2), with or without combination chemotherapy (5-FU + OXA). **I** Tumor images of HCT116 xenografts under different treatments (*N* = 10/group) (*left*). Tumor growth curves (*middle*) and quantitative analysis of final tumor weights (*right*). **J** Representative Ki-67 (*top*) and TUNEL (*bottom*) staining images for HCT116 xenograft model treated with nanoparticle-siRNAs and chemotherapy. *P < 0.05; **P < 0.01; ***P < 0.001; ****P < 0.0001

WNT/β-catenin is a key pathway involved in driving cancer stemness in CRC. In support of this notion, western blot confirmed that ADAT2 is positively correlated with stemness-related proteins, including LGR5, CD133, CD44, and EphB2 (Figure S9A). We thus tested whether ADAT2 modulates response of CRC cells to 5-Fluorouracil (5-FU) and Oxaliplatin (OXA). Measurement of 50% inhibitory concentration (IC_50_) values showed that ADAT2 overexpression in DLD1 and SW480 cells induced resistance to 5-FU and OXA and DLD1 and SW480 cells (Fig. 8A); whereas ADAT2 knockout in HCT116 cells increased their sensitivity to chemotherapy (Fig. 8B). Corroborating this, ADAT2 knockout in PDO74 organoids also enhanced the efficacy of 5-FU and OXA in suppressing growth (Fig. 8C) and induction of apoptosis (Fig. 8C and Figure S9B).

To validate the effect of ADAT2 in CRC chemoresistance in vivo, we investigated the effect of ADAT2 knockout plus chemotherapy (5-FU plus OXA) utilizing colon-specific ADAT2-cKO mice (Fig. 8D). As shown in Fig. 8E, ADAT2-cKO or 5-FU + OXA (in WT mice) partially suppressed tumor growth both in terms of tumor size (~ 60% reduction) and tumor number (~ 20%). Notably, 5-FU + OXA administration in ADAT2-cKO mice most strongly inhibited tumor growth, with > 90% inhibition in tumor volume and significantly decreased tumor number compared to control and single-treatment groups (Fig. 8F). Consistent with reduction in CRC tumorigenesis, the combination of ADAT2-cKO plus 5-FU + OXA most effectively suppressed cell proliferation (Ki-67) and promoted apoptosis (TUNEL) of CRC tumors (Fig. 8G).

We next tested the effect of ADAT2 knockout or overexpression on chemotherapy efficacy in CRC xenografts. In HCT116 xenografts, ADAT2 knockout plus 5-FU + OXA suppressed tumor growth compared to control, ADAT2 knockout, or 5-FU + OXA groups (Figure S9C), achieving tumor stasis. Accordingly, a combination of ADAT2 knockout plus 5-FU + OXA more effectively inhibited cell proliferation and enhanced apoptosis to a greater extent than either alone (Figure S9D). On the contrary, ADAT2 overexpression in DLD1 xenografts significantly limited efficacy of 5-FU + OXA (Figure S9E), and antagonized 5-FU + OXA-induced apoptosis and proliferative arrest (Figure S9F). These findings collectively infer that targeting ADAT2 is a plausible approach to improve chemotherapy efficacy in CRC.

### Targeting ADAT2 by VNP-siADAT2 synergizes with chemotherapy to suppress CRC growth in vivo

Finally, we constructed VNPs for in vivo delivery of ADAT2-targeting siRNA (Fig. 8H). Mice were injected with VNP-siNC or VNP-siADAT2, with or without chemotherapy (5-FU + OXA). Although either VNP-siADAT2 or 5-FU + OXA suppressed tumor growth, the combination of VNP-siADAT2 plus 5-FU + OXA treatment led to further reduction in tumor growth (Fig. 8I). The drug combination most effectively inhibited cell proliferation and promoted apoptosis (Fig. 8J), indicating that in vivo siADAT2 enhances chemotherapy efficacy. To assess the safety profile of VNP-siADAT2, we monitored body weight and performed blood biochemistry and histopathological analysis at the study endpoint. Mice treated with VNP-siADAT2 showed no significant loss in body weight compared to VNP-siNC group or untreated mice (Figure S10A). Indicators of hepatic and renal function remained within the normal range in all treatment groups (Figure S10B). Histological examination of major organs (liver, kidney, spleen, lung, heart) revealed no evidence of toxicity or tissue damage (Figure S10C). These data indicate that VNP-siADAT2 is well-tolerated in vivo.

## Discussion

Dysregulation of the epitranscriptome is a hallmark of cancer, but the functional impact of tRNA modifications on CRC remains understudied. Our comprehensive profiling of tRNA modifications unraveled A-to-I editing as the top up-regulated modification in CRC tumors. In support of this observation, ADAT2, the catalytic subunit involved in tRNA A-to-I conversion, is overexpressed in CRC patients and predicts poor prognosis, inferring its potential oncogenic function in colorectal tumorigenesis. A series of in vitro and in vivo models established that ADAT2 functions to drive tumorigenesis and chemoresistance in CRC, an effect mediated through codon usage-biased translational reprogramming. Taken together, ADAT2-mediated tRNA A-to-I modification promotes CRC development, and is a potential therapeutic target for boosting chemotherapy efficacy.

We explored the function of ADAT2 in colorectal tumorigenesis by generating conditional, intestine-specific ADAT2 knockout mice, showing that the depletion of ADAT2 impeded carcinogen-induced CRC. The effect of ADAT2 knockout was independently validated in mutant Apc-driven CRC, providing genetic evidence for the function of ADAT2 in CRC development. Corroborating these observations, knockout of ADAT2 in CRC cell lines and CRC organoids significantly impaired cell viability, migration and invasion, whilst ADAT2 overexpression exerted opposite functions. Although a number of reports have suggested that mRNA A-to-I modification, mediated by the double-stranded RNA-specific adenosine deaminase (ADARs), is involved in tumorigenesis and modulation of the tumor immune microenvironment [14], much less is known regarding the role A-to-I modifications in tRNAs in cancer. A recent study indicated that the requirement for tRNA A-to-I for a subset of genes encoding for low-complexity proteins important for cell adhesion [18]; however, their potential link to tumorigenesis is unclear. Our findings thus offered the first evidence of the tumor-promoting role of ADAT2 in CRC.

We next delineated molecular mechanism of ADAT2 in CRC. Domain-focused CRISPR/ Cas9 and mutagenesis studies identified that the deaminase domain, which catalyzes A-to-I modification in tRNAs, as essential for its pro-tumorigenic function. Theoretically, A-to-I modification are present in eight distinct tRNAs: tRNA^Leu^_AAG_, tRNA^Arg^_ACG_, tRNA^Ser^_AGA_, tRNA^Pro^_AGG_, tRNA^Ala^_AGC_, tRNA^Val^_AAC_, tRNA^Ile^_AAT_, and tRNA^Thr^_AGT_ [18, 19], necessary for the efficient translation of genes abundant in ADAT2-responsive codons [20–22]. A limited supply of A-to-I modified cognate tRNAs for mRNAs enriched in corresponding codons serves to constrain the rate of translation efficiency [23, 24], and could benefit from overexpression of ADAT2. Interestingly, tRNA-seq revealed that the manipulation of ADAT2 in CRC cells preferentially drives A-to-I conversion of tRNA^Leu^_AAG_, tRNA^Arg^_ACG_, and tRNA^Ser^_AGA_, implying cancer-specific promotion of translation with distinct codon bias. To pinpoint the downstream targets of ADAT2 relevant to the context of CRC, we performed integrated tRNA-seq, Ribo-seq, and RNA-seq analyses. We found that ADAT2 promotes an oncogenic translation program, principally involving upregulated translation of WNT/β-catenin pathway mediators. Considering codon usage bias towards the three critical tRNAs (tRNA^Leu^_AAG_, tRNA^Arg^_ACG_, tRNA^Ser^_AGA_), HDAC7 was identified as a downstream factor whose increased translation depends on ADAT2 in CRC. ADAT2-driven HDAC7 translation was validated by the increased translation efficiency, polysome-associated mRNA, and protein expression. Of note, HDAC7 mRNA displayed significantly elevated translation efficiency compared to a modified HDAC7 mRNA in which ADAT2-dependent codons were replaced with synonymous codons that do not require this modification for efficient decoding in ADAT2-overexpressing cells, suggesting that ADAT2 up-regulation in tumors might allow for the aberrant translation of codon-biased transcripts to support oncogenic phenotypes.

Supporting HDAC7 as a functional target of ADAT2 in colorectal tumorigenesis, HDAC7 knockout abrogated pro-tumorigenic effect of ADAT2 in CRC cells. Reciprocally, ectopic expression of HDAC7 restored cell proliferation in ADAT2-depleted cells. HDAC7, a class IIa deacetylase, is known to promote cancer progression via non-canonical deacetylation of non-histone proteins such as STAT3 and β-catenin [25, 26]. Indeed, pulldown and mass spectrometry assay revealed β-catenin as a top candidate interacting with HDAC7 in CRC cells, and it functions downstream of ADAT2 to promote the activation of WNT/β-catenin pathway, thereby driving the expression of oncogenic WNT target genes. This functional connection is supported by prior evidence showing that HDAC7 directly deacetylates β-catenin, facilitating its nuclear entry and transcriptional activity [27]. Corroborating our in vitro findings, HDAC7 and active β-catenin levels were simultaneously down-regulated in colon tumors from intestine-specific ADAT2 knockout mice. Furthermore, the expression of ADAT2 is positively correlated to that of HDAC7 and active β-catenin in CRC patients. In agreement with our data, HDAC7 overexpression in gastrointestinal cancers correlates with poor prognosis and therapy resistance [28–31]. Collectively, these findings define an ADAT2-HDAC7-WNT/β-catenin axis (Figure S11) as a signaling axis through which epitranscriptomic tRNA modifications promote tumor progression in CRC.

We further determined the therapeutic relevance of targeting ADAT2 in CRC. ADAT2 was found to increase the expression of WNT-associated stemness factors, including LGR5 and CD133, inferring its potential function in promoting cancer stemness and chemoresistance. We thus evaluated ADAT2 as a therapeutic target and found that sgADAT2 potentiated the effect of standard-of-care chemotherapies (5-FU or oxaliplatin) in inhibiting CRC growth in vitro, and that intestine-specific ADAT2 knockout in mice in combination with 5-FU plus oxaliplatin achieved > 90% reduction in tumor load in vivo. Moreover, a nanoparticle-siADAT2 formulation also improved chemotherapy efficacy against CRC xenografts. This observation implies that targeting ADAT2 overcomes chemoresistance, offering a potential combinatorial strategy for treatment of CRC patients. Translational impact of our findings is strengthened by clinical data showing that elevated ADAT2 expression in CRC served as an independent prognostic factor associated with poor survival. Hence, ADAT2 might be a useful biomarker for stratifying patients according to their survival outcomes.

Our study has several potential limitations. First, while we elucidated the oncogenic role of ADAT2 in CRC, its potential effects on the tumor microenvironment remain unexplored. Furthermore, to establish the causal role of ADAT2 overexpression in tumor initiation, the development of conditional, intestine-specific ADAT2 knock-in transgenic mouse models is warranted. Finally, while our proof-of-concept study using VNP-siADAT2 supports the potential of ADAT2 as a therapeutic target, future studies using ADAT2-specific inhibitors will be crucial to define the safety and therapeutic index of ADAT2 inhibition.

In conclusion, we have uncovered ADAT2 as a regulator of oncogenic translation in CRC. ADAT2 promotes colorectal tumorigenesis and chemoresistance by activating an ADAT2-HDAC7-WNT/β-catenin axis. This is achieved by tRNA A-to-I modifications that mediate codon usage-biased translational reprogramming, leading to amplified synthesis of tumor-promoting proteins such as HDAC7. Our findings nominate ADAT2 as a therapeutic target for CRC treatment and as a prognostic biomarker.

## Supplementary Information

Supplementary Material 1.

Supplementary Material 2.

Supplementary Material 3.

## Data Availability

All data is available within the article and its supplementary materials.
