# Supplementary material for: ADAT2-mediated A-to-I tRNA modification promotes oncogenic translation and colorectal cancer progression and chemoresistance
Source: Mol Cancer. 2026 Mar 17;25:118. doi: 10.1186/s12943-026-02618-5 (PMC13107624; doi:10.1186/s12943-026-02618-5)
Supplement: Supplementary file 1 — Supplementary Material 1. [file 12943_2026_2618_MOESM1_ESM.pdf]

**Table S1. Primer sequences**

| Real-time PCR | Human Primers |
|---|---|
| *ADAT2*- F | CGACATGCAGAAATGGTGGC |
| *ADAT2*- R | ACCAGCGGGATTTTCATCAGG |
| *GAPDH*- F | GCACCACCAACTGCTTAGCA |
| *GAPDH*- R | TCTTCTGGGTGGCAGTGATG |
| *β-ACTIN*- F | CTCACCATGGATGATGATATCGC |
| *β-ACTIN*- R | GGAATCCTTCTGACCCATGCC |
| *HDAC7*- F | CTCTCGCCGTCTCACAGTC |
| *HDAC7*- R | CTCACCTGGGTCCCATCAGC |
| *Luciferase*- F | AAGTAGTGTGTGCCCGTCTG |
| *Luciferase*- R | CCTCTGGTTTCCCTTTCGCT |
| *KRT7*- F | GCCAGGGAAACTAGCTGGG |
| *KRT7*- R | AGAACCTAGAGATCCCGCTC |
| *SPHK1*- F | GGTTATGGATCCAGTGGTCGG |
| *SPHK1*- R | CCCGCTGAGAAAACAAAACCA |
| *ATP6V1E2*- F | ATTTCACCTCCACCCGGAAG |
| *ATP6V1E2*- R | AGTTCAGGCCTCCCTTTTGG |
| *SLCO4A1*- F | GAGACGGGGACGGACACA |
| *SLCO4A1*- R | CTGATGCAGGGGCATCTCC |
| *WNT7B*- F | GGCCCACCATGCTTCTACTG |
| *WNT7B*- R | CCACGGATGACAGTGCTAAGT |
| *ZDHHC1*- F | CGGATCCGACCCCAGAAAAA |
| *ZDHHC1*- R | GAATCCGTCGACGAGCTGGAG |

**Table S2. Antibody list**

| Antibody | Vendor | Cat no. | Dilution for WB | Dilution for IHC | Dilution for IF |
|---|---|---|---|---|---|
| ADAT2 | Abbexa | abx230153 | 1:500 | 1:100 | 1:100 |
| HDAC7 | Cell signaling | 33418 | 1:1000 | 1:100 | |
| Non-phospho (Active) β-Caten | Cell signaling | 8814 | 1:1000 | 1:500 | |
| β-Catenin | Cell signaling | 8480 | 1:1000 | | |
| Ki-67 | Cell signaling | 12202 | | 1:500 | |
| Vimentin | Cell signaling | 5741 | 1:1000 | | |
| E-cadherin | Cell signaling | 14472 | 1:1000 | | |
| Cleave caspase7 | Cell signaling | 9491 | 1:500 | | |
| Caspase7 | Cell signaling | 9492 | 1:1000 | | |
| Cleave PARP | Cell signaling | 5625 | 1:1000 | | |
| PARP | Cell signaling | 9532 | 1:1000 | | |
| PCNA | Cell signaling | 13110 | 1:1000 | | |
| Cyclin D1 | Cell signaling | 2922 | 1:1000 | | |
| c-Myc | Cell signaling | 9402 | 1:1000 | | |
| Lamin A/C | Cell signaling | 4777 | 1:1000 | | |
| LGR5 | Origene | TA503316 | 1:1000 | | |
| CD133 | Cell signaling | 64326 | 1:1000 | | |
| CD44 | Cell signaling | 5640 | 1:1000 | | |
| EphB2 | Cell signaling | 83029 | 1:1000 | | |
| Puromycin | Sigma-Aldrich | 12D10 | 1:25,000 | | |
| GAPDH | Cell signaling | 5174 | 1:1000 | | |
| β-actin | Cell signaling | 4970 | 1:1000 | | |
| IgG (rabbit) | Sigma-Aldrich | 12-370 | | | |

**Table S3. Oligonucleotide sequences**

| Name | Sequences |
|---|---|
| sgRNA#1 targeting *ADAT2* | GTCTACAACAATGAAGTTGT |
| sgRNA#2 targeting *ADAT2* | GAAAATACTGAAGTTCCTGT |
| sgRNA#3 targeting *ADAT2* | GCTTCCTCAGCCCGATATCC |
| sgRNA targeting *HDAC7* | GCGCTCGGTGGAGCCCATGA |
| siRNA#1 targeting *ADAT2* | sense: 5'-GCUGUCAGAAUGAACGAUUTT-3' |
| | anti-sense: 5'-AAUCGUUCAUUCUGACAGCTT-3' |
| siRNA#2 targeting *ADAT2* | sense: 5'-GCAGUGGAAAUGUUAAAGATT-3' |
| | anti-sense: 5'-UCUUUAACAUUUCCACUGCTT-3' |
| siRNA#3 targeting *ADAT2* | sense: 5'-GUCCCUCUGAAGUAUUUGATT-3' |
| | anti-sense: 5'-UCAAAUACUUCAGAGGGACTT-3' |
| siRNA#4 targeting *ADAT2* | sense: 5'-GCUGUCUUAUGGUCUACAATT-3' |
| | anti-sense: 5'-UUGUAGACCAUAAGACAGCTT-3' |
| siRNA#5 targeting *ADAT2* | sense: 5'-ACUCGACAUUCAGUGAUGGTT-3' |
| | anti-sense: 5'-CCAUCACUGAAUGUCGAGUTT-3' |

**Table S4. Sequences of ADAT2 sgRNAs for CRISPR domain–targeting assay**

| Name | Forward (5'–3') | Reverse (5'–3') |
|---|---|---|
| sg-1 | GCACCCAAGCCAGCTGCAAG | AAACCTTGCAGCTGGCTTGGGTGC |
| sg-2 | CGCGTGCTCGGTGTCGGCAG | AAACCTGCCGACACCGAGCACGCG |
| sg-3 | AGAGGAGACCGAAAAGTGGA | AAACTCCACTTTTCGGTCTCCTCT |
| sg-4 | GATGGAGGAGGCGATGCACA | AAACTGTGCATCGCCTCCTCCATC |
| sg-5 | AGTATTTTCGAGGGCTTCTT | AAACAAGAAGCCCTCGAAAATACT |
| sg-6 | AGTTCCTGTTGGCTGTCTTA | AAACTAAGACAGCCAACAGGAACT |
| sg-7 | GTCTACAACAATGAAGTTGT | AAACACAACTTCATTGTTGTAGAC |
| sg-8 | TACTCGACATGCAGAAATGG | AAACCCATTTCTGCATGTCGAGTA |
| sg-9 | CCATCGATCAGGTCCTCGAT | AAACATCGAGGACCTGATCGATGG |
| sg-10 | CACTTTGACGACACCAATCG | AAACCGATTGGTGTCGTCAAAGTG |
| sg-11 | GTGTGTTCAAATACTTCAGA | AAACTCTGAAGTATTTGAACACAC |
| sg-12 | CACTGTGTTGTATGTCACTG | AAACCAGTGACATACAACACAGTG |
| sg-13 | GCAGCTGCACACATAATGCA | AAACTGCATTATGTGTGCAGCTGC |
| sg-14 | GACAGCCATATACAACCAGC | AAACGCTGGTTGTATATGGCTGTC |
| sg-15 | AATGAACGATTTGGTGGTTG | AAACCAACCACCAAATCGTTCATT |
| sg-16 | AGTGTTTGGTAGGTCAGCAG | AAACCTGCTGACCTACCAAACACT |
| sg-17 | TGAAATGGTCTCCCAGTGTT | AAACAACACTGGGAGACCATTTCA |
| sg-18 | TATCCCTGGATATCGGGCTG | AAACCAGCCCGATATCCAGGGATA |
| sg-19 | AAAATCGAAAGTTCGGAAAA | AAACTTTTCCGAACTTTCGATTTT |
