# Supplementary material for: ADAT2-mediated A-to-I tRNA modification promotes oncogenic translation and colorectal cancer progression and chemoresistance
Source: Mol Cancer. 2026 Mar 17;25:118. doi: 10.1186/s12943-026-02618-5 (PMC13107624; doi:10.1186/s12943-026-02618-5)
Supplement: Supplementary file 2 — Supplementary Material 2. [file 12943_2026_2618_MOESM2_ESM.pdf]

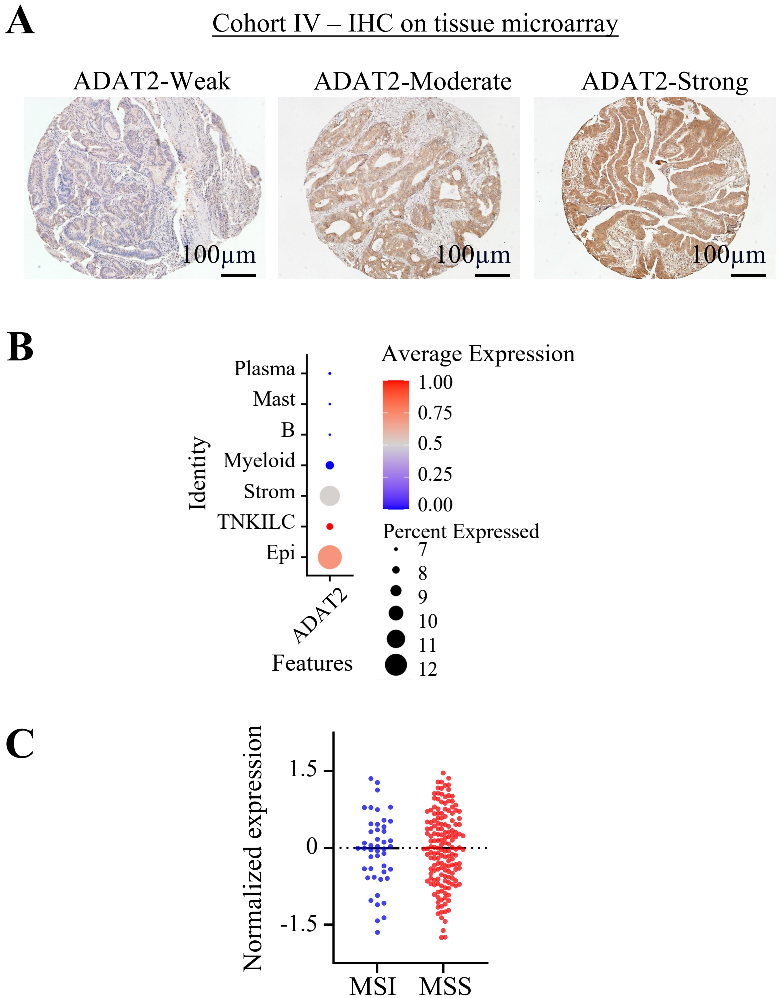

Figure S1. ADAT2 expression in CRC.

(A) Representative IHC of ADAT2 protein expression in a subset of paired samples from Cohort IV.

(B) Single-cell analysis of ADAT2 expression in CRC tissues (GSE178341).

(C) Analysis of ADAT2 mRNA expression in the TCGA-COAD microarray cohort (AgilentG4502A_07_3) comparing microsatellite stable (MSS, N=163) and microsatellite instable (MSI, includes MSI-H and MSI-L, N=46) tumors.

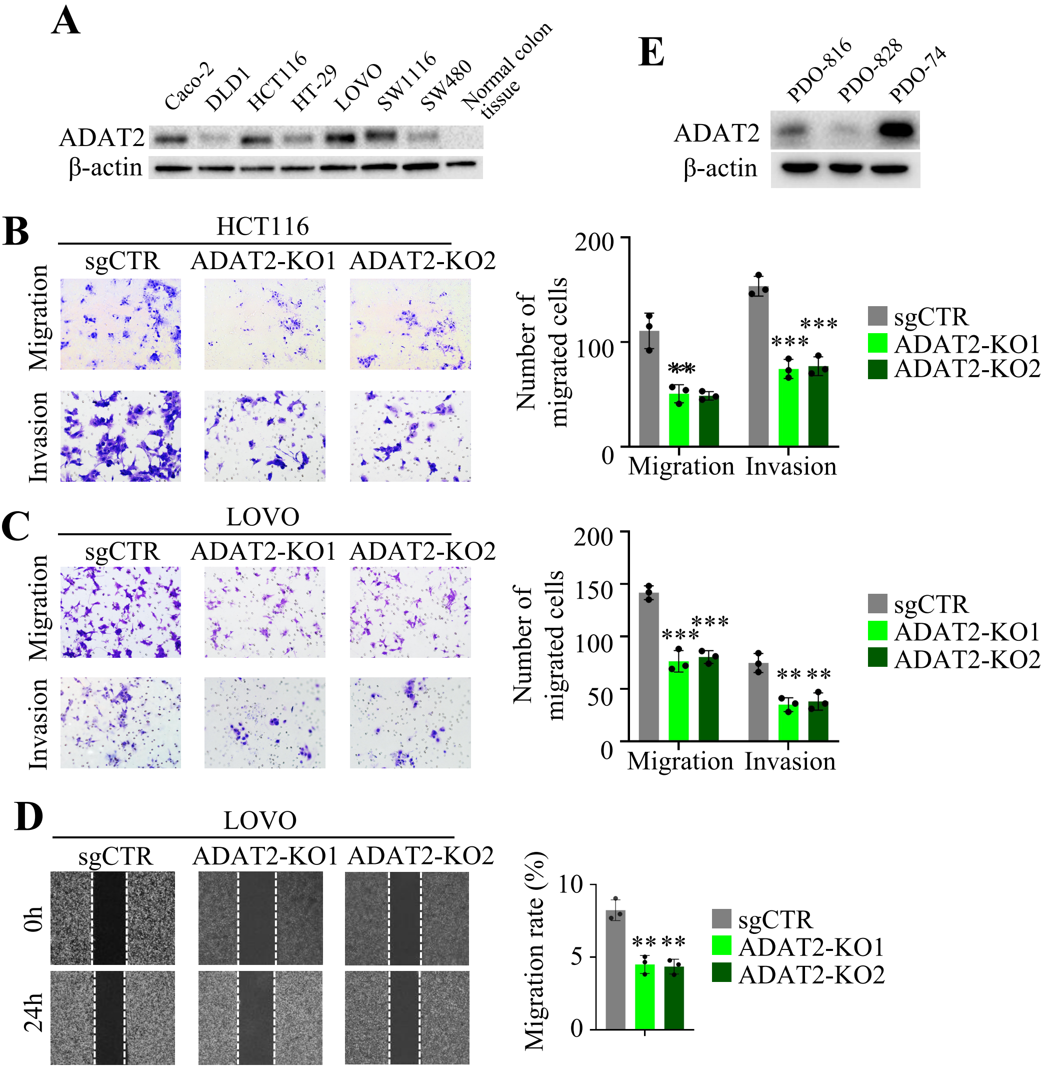

Figure S2. ADAT2 drives pro-metastatic phenotypes in CRC.

(*A*) ADAT2 protein expression in CRC cell lines by western blot.

(*B*) Transwell migration and Matrigel invasion assays of control and ADAT2-knockout HCT116 cells (*left*). Statistical analysis (N=3) (*right*).

(*C*) Transwell migration and Matrigel invasion assays of control and ADAT2-knockout LOVO cells (*left*). Statistical analysis (N=3) (*right*).

(D) Wound healing assay of control and ADAT2-knockout LOVO cells (*left*). Statistical analysis (N=3) (*right*).

(*E*) Western blot analysis of ADAT2 protein expression in CRC patient-derived organoids.

*P<0.05; **P<0.01; ***P<0.001.

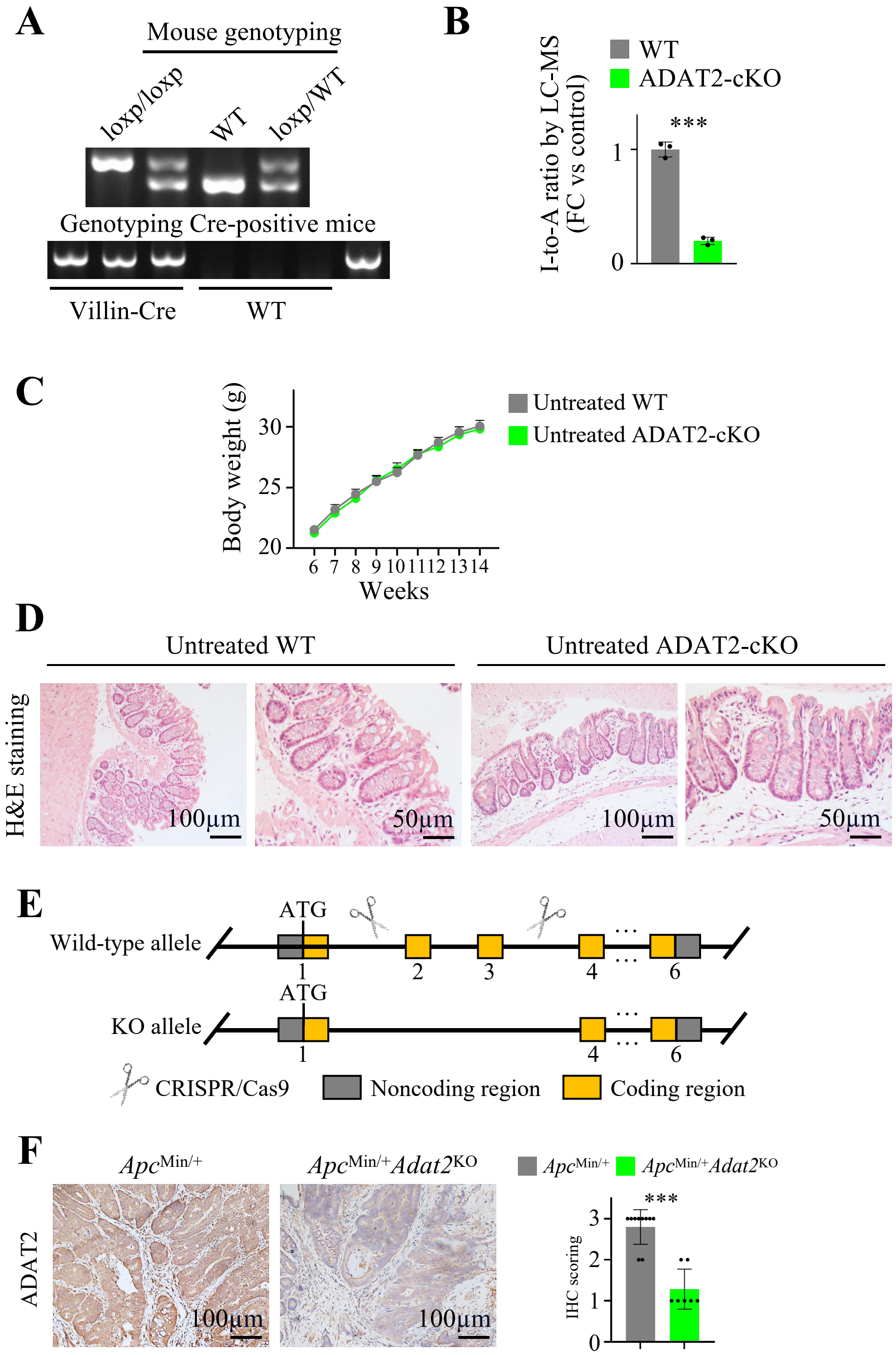

Figure S3. Genetic ablation of ADAT2 suppresses colorectal tumorigenesis *in vivo*.

(A) PCR genotyping confirmed the presence of the floxed *Adat2* allele and *Villin-Cre* transgene.

(B) LC-MS analysis of I-to-A ratio in colon tissues from ADAT2 cKO mice (N=3) and wildtype littermates (N=3).

(C) Body weight analysis of ADAT2 cKO mice and wildtype littermates (N=3).

(D) Histological assessment of normal colon tissues from untreated wildtype and untreated ADAT2 cKO mice.

(E) Schematic for whole body Adat2-KO mice.

(F) Immunohistochemistry analysis of ADAT2 expression in colon tumors from *Apc*$^{Min/+}$*ADAT2*^{KO} (N = 7) mice compared with controls (N = 10).

**P*<0.05; ***P*<0.01; ****P*<0.001.

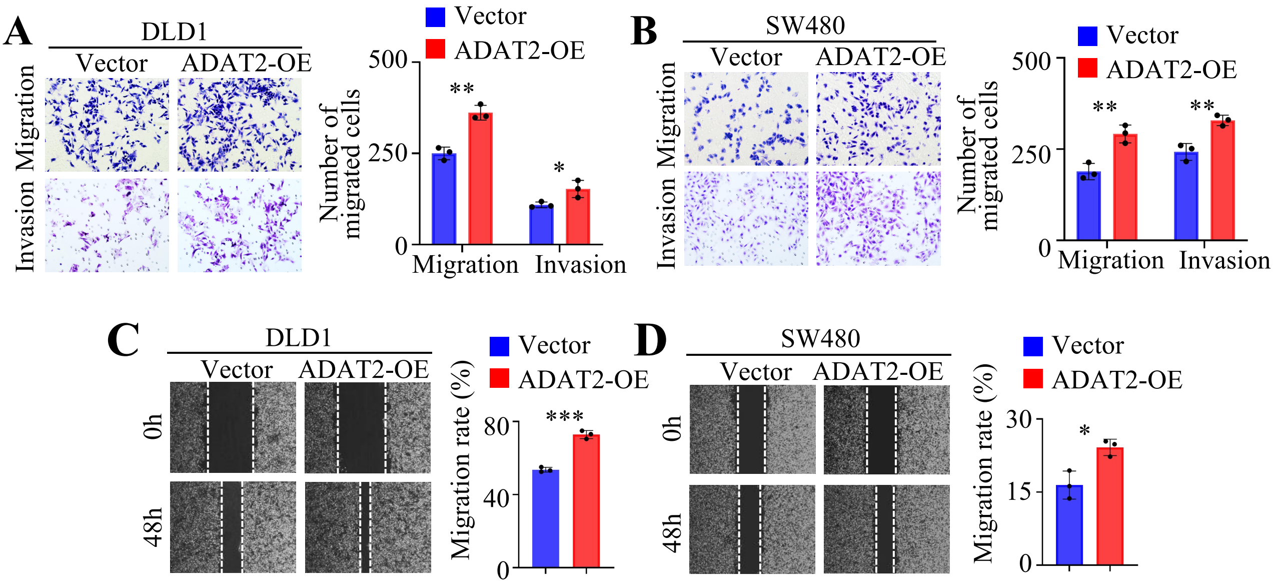

Figure S4. ADAT2 overexpression promotes pro-metastatic phenotypes in CRC cells.

(A) Transwell migration and Matrigel invasion assays of control and ADAT2-overexpressing DLD1 cells (*left*). Statistical analysis (N=3) (*right*).

(B) Transwell migration and Matrigel invasion assays of control and ADAT2-overexpressing SW480 cells (*left*). Statistical analysis (N=3) (*right*).

(C) Wound healing assay of control and ADAT2-overexpressing DLD1 cells (*left*). Statistical analysis (N=3) (*right*).

(D) Wound healing assay of control and ADAT2-overexpressing SW480 cells (*left*). Statistical analysis (N=3) (*right*).

*$P<0.05$; **$P<0.01$; ***$P<0.001$.

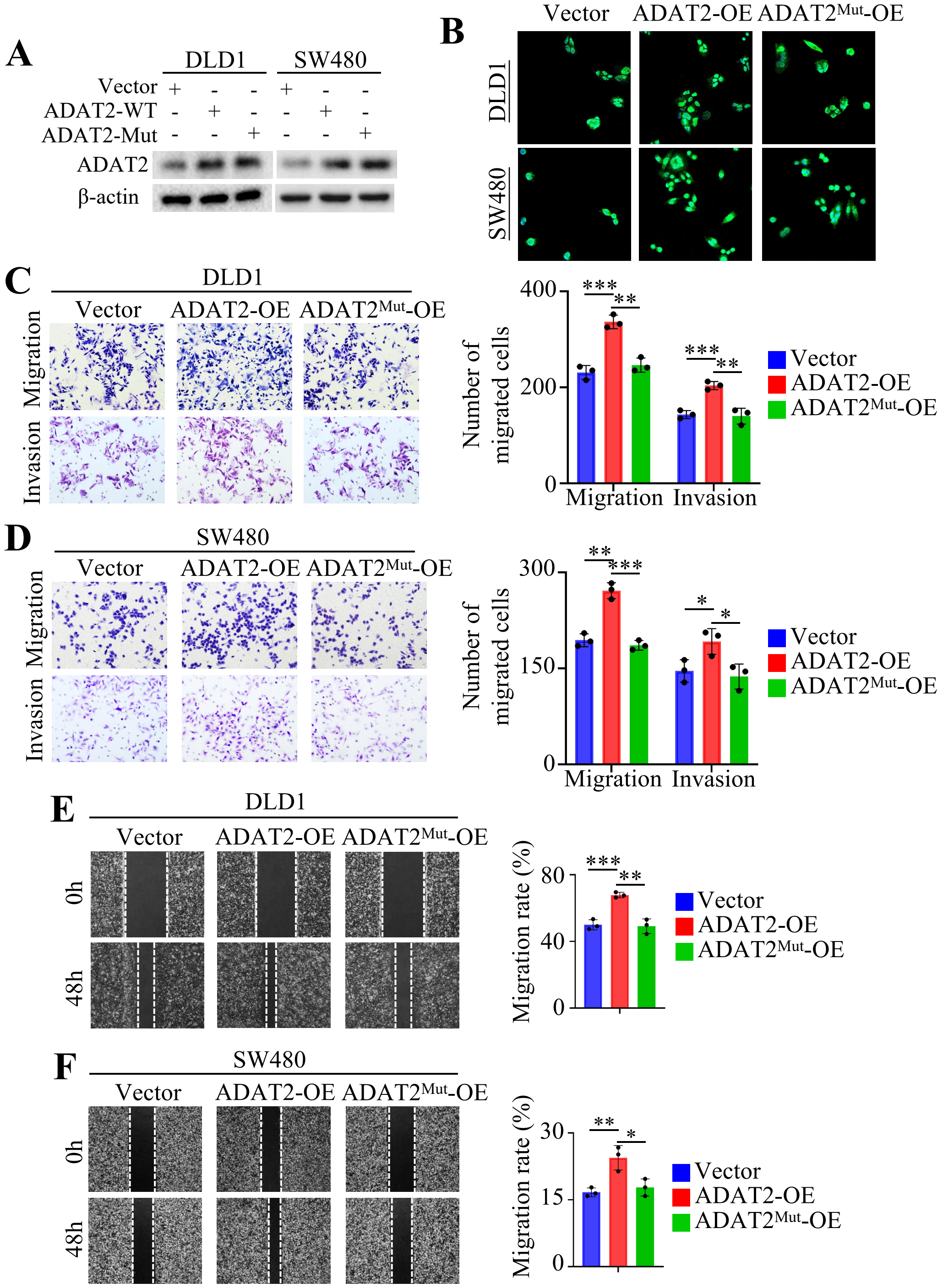

Figure S5. ADAT2 A-to-I catalytic activity is required for its pro-metastatic function.
(A) Western blot validated overexpression of ADAT2-WT and catalytically-dead ADAT2 mutant in DLD1 and SW480 cells.
(B) Subcellular localization of wild-type and catalytically dead ADAT2.
(C) Transwell migration and Matrigel invasion assays of control, ADAT2-WT-overexpressing and ADAT2 mutant-overexpressing DLD1 cells (*left*). Statistical analysis (N=3) (*right*).
(D) Transwell migration and Matrigel invasion assays of control, ADAT2-WT-overexpressing and ADAT2 mutant-overexpressing SW480 cells (*left*). Statistical analysis (N=3) (*right*).
(E) Wound healing assay of control, ADAT2-WT-overexpressing and ADAT2 mutant-overexpressing DLD1 cells (*left*). Statistical analysis (N=3) (*right*).
(F) Wound healing assay of control, ADAT2-WT-overexpressing and ADAT2 mutant-overexpressing SW480 cells (*left*). Statistical analysis (N=3) (*right*).
$^*P<0.05$; $^{**}P<0.01$; $^{***}P<0.001$.

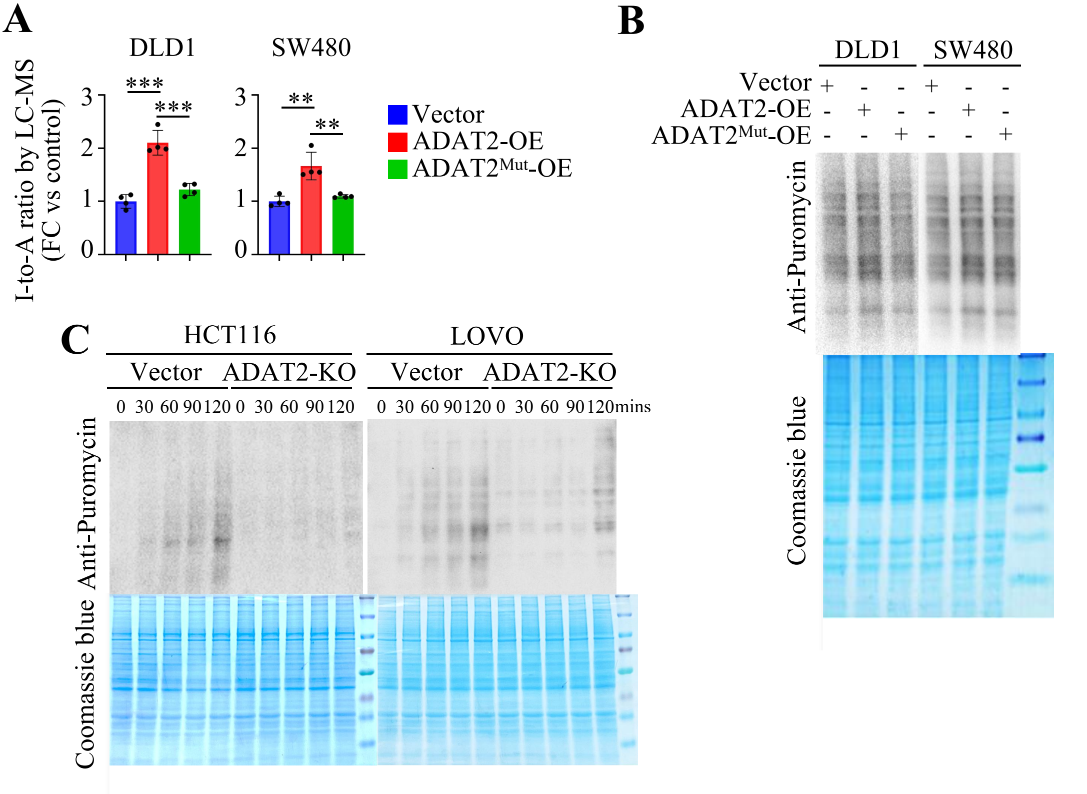

Figure S6. ADAT2 promotes A-to-I tRNA modification-dependent translation in CRC cells.

(A) LC-MS analysis of I-to-A ratio in control, ADAT2-WT-overexpressing, and catalytically-dead ADAT2 mutant-overexpressing DLD1 and SW480 cells (N=3).

(B) Puromycin incorporation assay in cells with overexpression of ADAT2-WT or ADAT2 mutant. Immunoblotting with anti-puromycin antibody showed the levels of newly synthesized proteins.

(C) Puromycin incorporation assay in control and ADAT2-knockout HCT116 and LOVO cells. Immunoblotting with anti-puromycin antibody showed the levels of newly synthesized proteins.

***P*<0.01; ****P*<0.001.

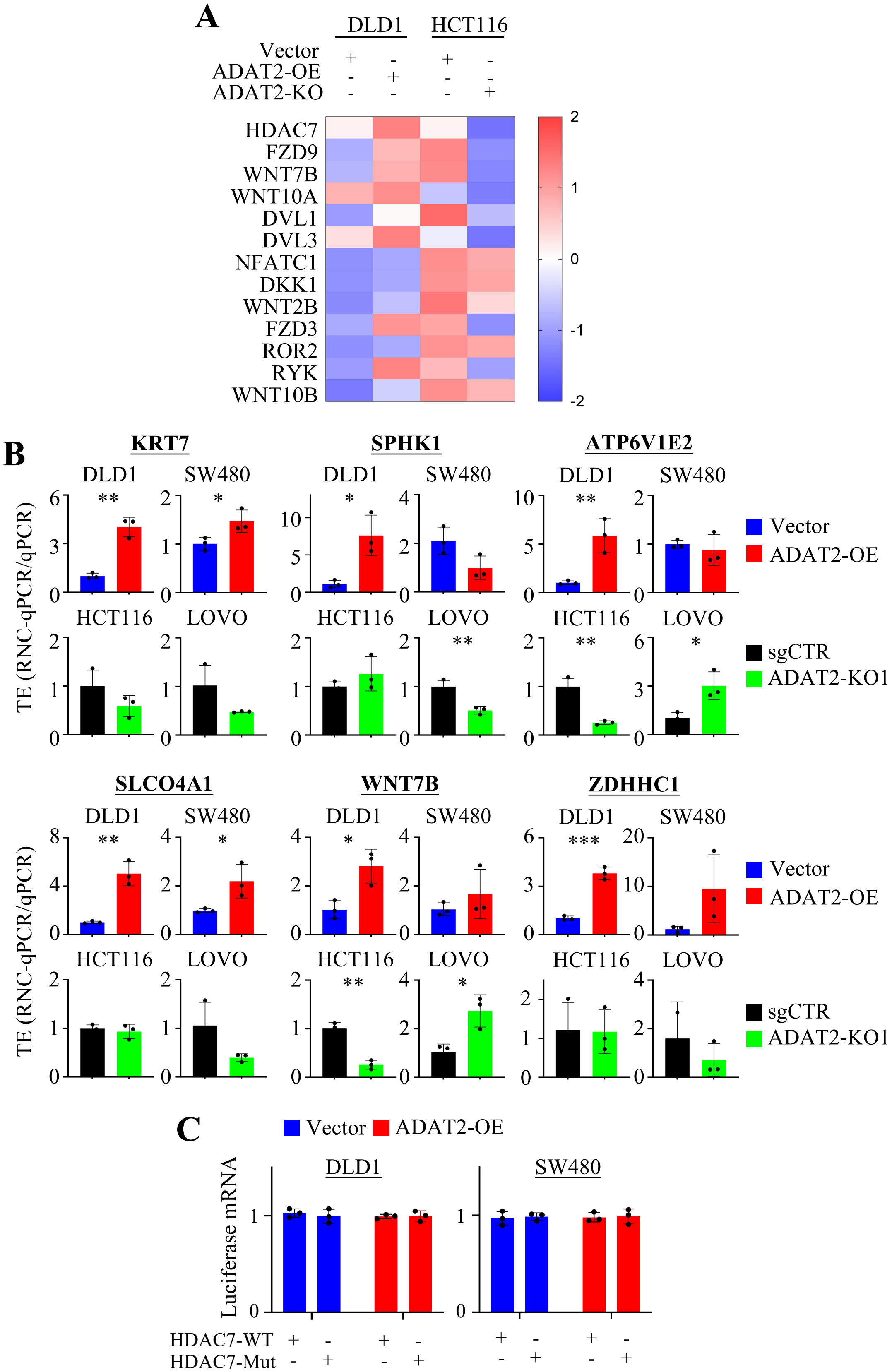

Figure S7. Downstream targets of ADAT2 in CRC.

(A) Heatmap of Ribo-seq data showing the effect of ADAT2 overexpression and knockout on the translation of WNT pathway genes.

(B) Translation efficiency (RNC-qPCR to qPCR ratio) of other candidate genes, ATP6V1E2, KRT7, SLCO4A1, SPHK1, WNT7B, and ZDHHC1. (N=3)

(C) Quantitative RT-PCR analysis of luciferase mRNA derived from wildtype (WT) and codon-mutated (Mut) HDAC7 reporters. (N=3)

*P<0.05; **P<0.01.

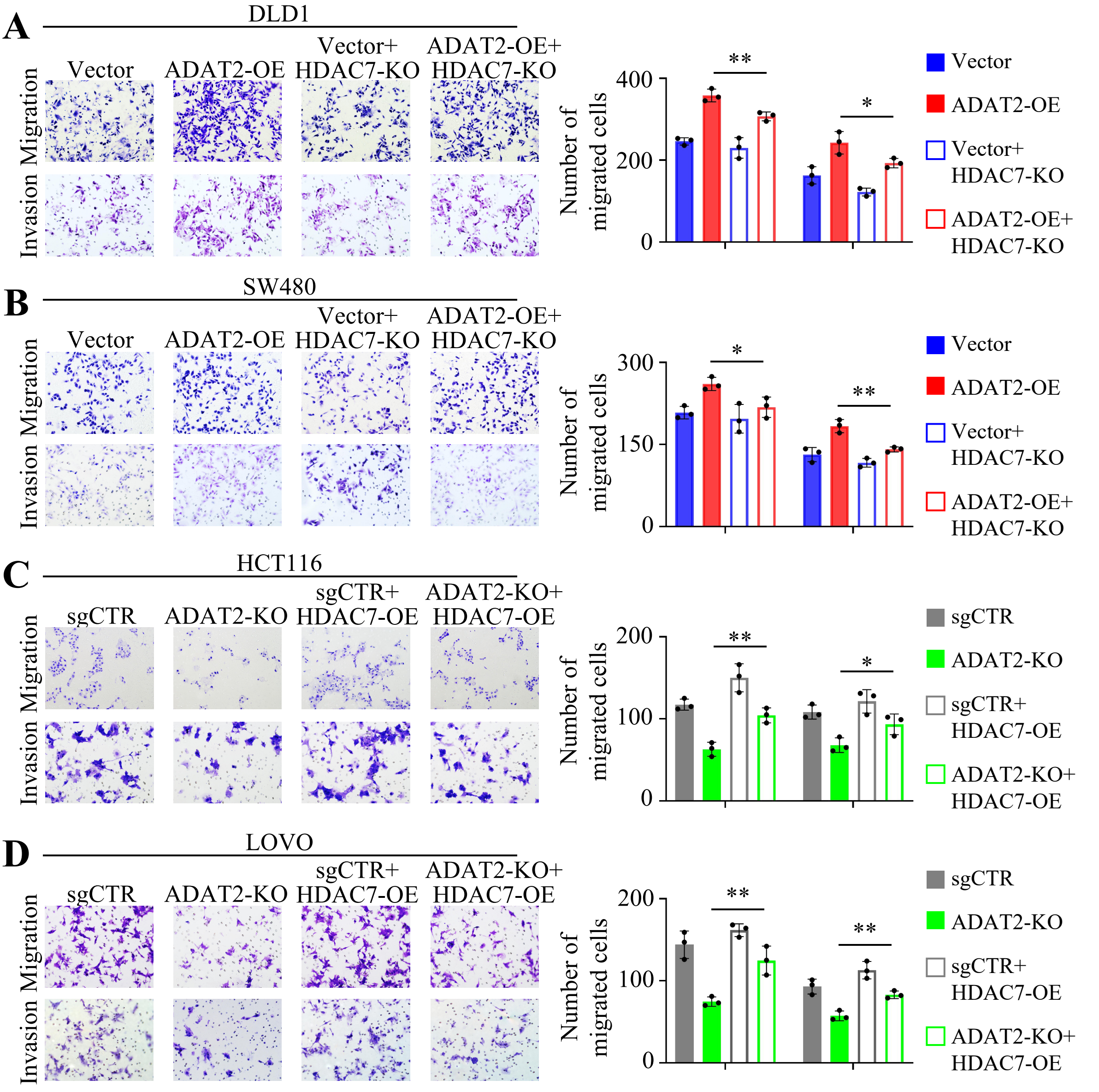

Figure S8. ADAT2 promotes pro-metastatic phenotypes through HDAC7.
(*A*) Transwell migration and Matrigel invasion assays of control and ADAT2-OE DLD1 cells, with or without HDAC7 knockout (*left*). Statistical analysis (N=3) (*right*).
(*B*) Transwell migration and Matrigel invasion assays of control and ADAT2-OE SW480 cells, with or without HDAC7 knockout (*left*). Statistical analysis (N=3) (*right*).
(*C*) Transwell migration and Matrigel invasion assays of control and ADAT2-KO HCT116 cells, with or without HDAC7 overexpression (*left*). Statistical analysis (N=3) (*right*).
(*D*) Transwell migration and Matrigel invasion assays of control and ADAT2-KO LOVO cells, with or without HDAC7 overexpression (*left*). Statistical analysis (N=3) (*right*).
**P*<0.05; ***P*<0.01.

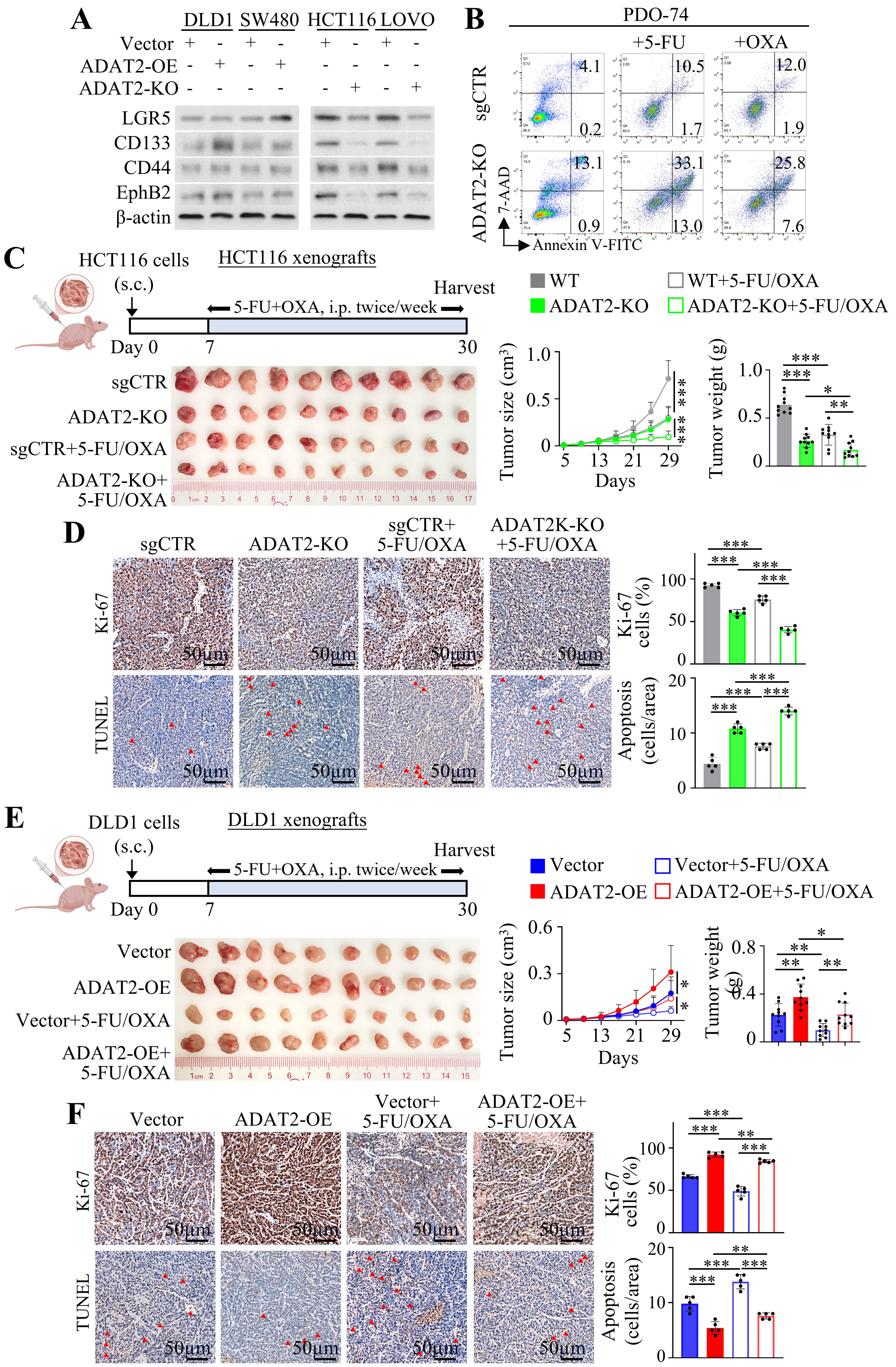

Figure S9. ADAT2 promotes stemness and chemoresistance in CRC.

(*A*) Western blot of stemness markers in ADAT2-overexpressing and knockout CRC cells.

(*B*) Flow cytometry analysis of apoptosis induced by ADAT2 knockout plus chemotherapy.

(*C*) HCT116 cells with or without ADAT2 knockout were subcutaneously injected into mice, followed by 5-FU+OXA treatment (*left*). Tumor growth curve and weight (N=10). (*right*).

(*D*) Ki-67 and TUNEL staining of HCT116 xenografts.

(*E*) DLD1 cells with or without ADAT2 overexpression were subcutaneously injected into mice, followed by 5-FU+OXA treatment (*left*). Tumor growth curve and weight (N=10). (*right*).

(*F*) Ki-67 and TUNEL staining of DLD1 xenografts.

*P<0.05; **P<0.01; ***P<0.001.

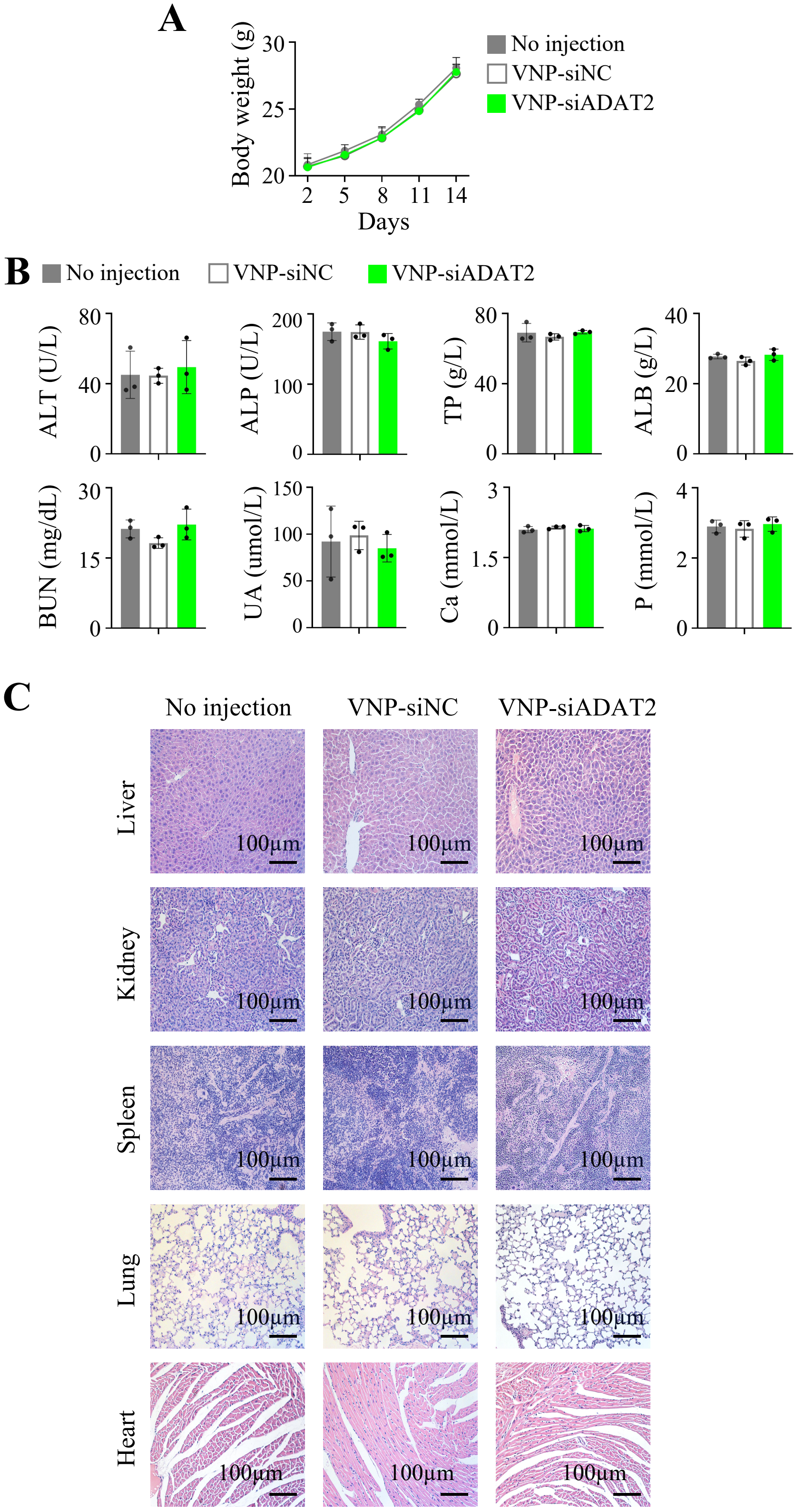

Figure S10. *In vivo* safety and toxicity assessment of VNP-siADAT2 therapy.

(A) Body weight curves for untreated, VNP-siNC, and VNP-siADAT2 groups (N=3).

(B) Plasma biochemistry analysis of hepatic and renal function (N=3).

(C) Representative histopathology of liver, kidney, spleen, lung, and heart tissues.

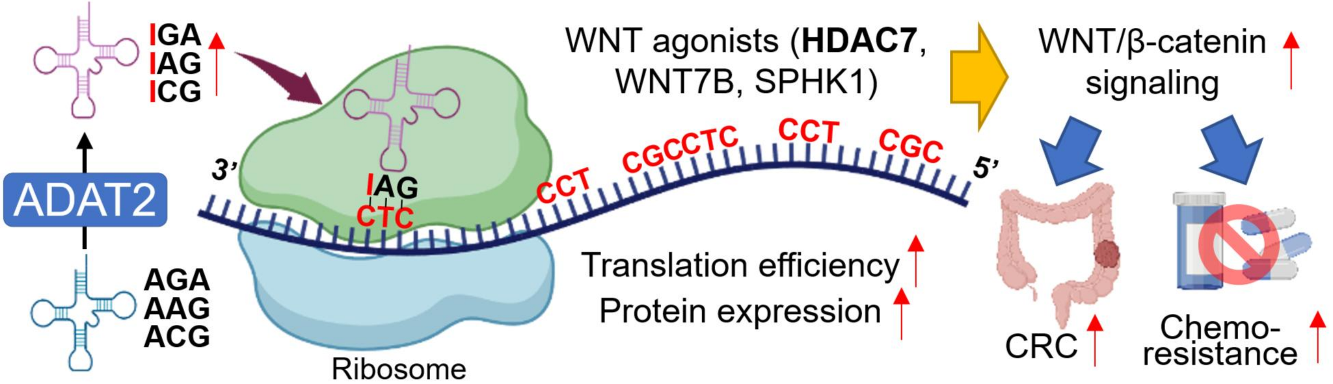

Figure S11. Schematic diagram showing the mechanism of action of ADAT2 in tumorigenesis and chemoresistance of CRC.
