# Supplementary material for: ADAT2-mediated A-to-I tRNA modification promotes oncogenic translation and colorectal cancer progression and chemoresistance
Source: Mol Cancer. 2026 Mar 17;25:118. doi: 10.1186/s12943-026-02618-5 (PMC13107624; doi:10.1186/s12943-026-02618-5)
Supplement: Supplementary file 3 — Supplementary Material 3. [file 12943_2026_2618_MOESM3_ESM.pdf]

## Supplemental Methods

### LC-MS/MS analysis of tRNA metabolites

Cells were washed by PBS three times, then total RNA was extracted using TRIzol (Thermo Fisher Scientific). To analyze tRNA modifications, tRNA was purified from total RNA by magnetic beads (BioDynami, Cat.# 40054M). This purification step specifically enriches tRNAs and removes the majority of other RNA species, including ribosomal RNA (rRNA), mRNA, and small non-coding RNAs, as well as free nucleosides. Subsequently, 200 ng of tRNA in 7 μl of water was heated at 95°C for 2 minutes and then chilled on ice for 30 seconds. The sample was then mixed with Nuclease P1 (New England Biolabs) in 1X Nucleoside Digestion buffer in a 20 μl system at 37°C for 18 hours. The enzyme was deactivated by heating at 90°C for 3 minutes. After centrifugation at 12,000 *g* for 10 minutes at 4°C, the supernatant was collected and diluted in deionized distilled water containing formic acid (0.1%, v/v) for LC-MS injection. Instrument analysis was carried on a Thermo Scientific UPLC system coupled to a TSQ Quantiva™ Triple Quadrupole MS equipped with an ESI source. A Waters HSS T3 column (2.1 mm ×100 mm, 1.8 μm) was employed for the separation. The column was kept at 35°C during the analysis. The mobile phases were water containing 0.01% FA (A) and acetonitrile (B). Mass spectrometry analysis was performed in negative and positive ion multiple reaction monitoring mode.

### tRNA purification

Total RNA samples were transferred to a 96-well plate, and 1X volumes of beads (BioDynami, USA) were added to the wells containing the samples. The sample plate was then placed on a magnet, and the supernatant was transferred to a new well without

disturbing the beads, while the bead pellets were discarded. Next, 1.5X volumes of beads were added to the wells containing the supernatants. 180 µl of 80% ethanol was added without disturbing the beads, and all residual ethanol was removed after incubation, again without disturbing the beads. Finally, the beads were air-dried on the magnet, the plate was removed from the magnet, and the beads were resuspended in 20 µl of water or Tris-HCl (10 mM). The plate was then reloaded on the magnet, and the supernatant (containing the sample) was transferred to a new tube without disturbing the beads.

**RNA isolation and RT-qPCR**

Total RNA was extracted using TRIzol reagent, and its concentration was determined using a NanoDrop (Thermo Fisher, USA). Complementary DNA was synthesized from total RNA by using the PrimeScript RT Reagent Kit (Takara, RR037B). Quantitative PCR (qPCR) was carried out using the One-Step TB Green PrimeScript RT-PCR Kit II (Takara, RR086B) on the ViiA7 Real-Time PCR System (Thermo Fisher Scientific) in either 96- or 384-well plate formats. Each assay was performed in triplicate, with ACTB serving as the internal control. Relative gene expression was calculated using the $2-\Delta\Delta CT$ method. The sequences of primers used in this study are provided in Supplementary Table S1.

**Human colorectal cancer and adjacent non-tumor tissue samples**

Four cohorts of patients with CRC were included in the study. Cohort I comprised data from The Cancer Genome Atlas (TCGA), which included ADAT2 mRNA expression from 380 CRC tissues and 51 normal tissues. Cohort II, sourced from Peking University Cancer Hospital, consisted of 150 CRC patients whose surgically excised tumor tissues and adjacent non-tumor tissues were collected. Cohort III was from the GSE100179 dataset,

which included 20 healthy colonic tissues and 20 CRC tissues. Cohort IV from the tissue microarray was composed of formalin-fixed, paraffin-embedded tissues from 157 patients with CRC, collected at Prince of Wales Hospital in Hong Kong. The research protocols received approval from the Clinical Research Ethics Committee at Prince of Wales Hospital, The Chinese University of Hong Kong, as well as from Peking University Cancer Hospital. All patients provided written informed consent for the collection of study specimens. This research was conducted in accordance with the Declaration of Helsinki established by the World Medical Association.

**Tissue microarray assay**

Archival formalin-fixed, paraffin-embedded tissue specimens from 157 patients with CRC who underwent colectomy at the same hospital between 1995 and 2014 were retrieved. All specimens were reviewed by an expert gastrointestinal pathologist to confirm histologic diagnosis and tumor cell content. Clinicopathologic information was retrieved from the hospital database, last updated in October 2025. Immunohistochemistry was performed on CRC tissue microarrays with ADAT2 (1:100 dilution, Abbexa no. abx230153), HDAC7 (1:100 dilution, CST no. 33418), and non-phospho (active) β-catenin (1:500 dilution, CST no. 8814) antibody. The staining scores of ADAT2, HDAC7, and non-phospho (active) β-catenin were determined by two pathologists independently. IHC score 1: <10% staining; IHC score 2: ≤70% weak or moderate staining or strong staining in 10%–30%; IHC score 3: moderate staining in >70% or strong staining in >30%.

**Protein extraction and western blot analysis**

Cells were lysed using the CytoBuster Protein Extraction Kit (Merck Millipore, 71009), which included protease inhibitors (Roche) and phosphatase inhibitors (Roche). Protein concentration was measured with the Pierce BCA Protein Assay Kit (Thermo Fisher Scientific, 23227). The proteins were then separated through 10-12% sodium dodecyl sulfate-polyacrylamide gel electrophoresis (SDS–PAGE) and subsequently transferred onto polyvinylidene difluoride (PVDF) membranes (BioRad). Following blocking with 5% bovine serum albumin (BSA), the blots were incubated with primary antibodies overnight at 4 °C, followed by incubation with secondary antibodies for 1 hour. Proteins of interest were detected utilizing the SuperSignal West Femto Maximum Sensitivity Substrate (Thermo Fisher Scientific) in conjunction with the ChemiDoc XRS+ System (Bio-Rad, Hercules, CA). β-actin or GAPDH served as the housekeeping controls.

**H&E and IHC assays**

For H&E (Hematoxylin and Eosin) staining, the paraffin-embedded sections (4 mm) were deparaffinized using xylene, then rehydrated through a gradient of ethanol. They were subsequently stained with hematoxylin for 6 minutes and eosin for 30 seconds. For IHC (Immunohistochemistry) assay, antigens were retrieved using sodium citrate buffer (10 mmol/L sodium citrate, 0.05% Tween 20, pH 6.0) after deparaffinization and rehydration. The sections were treated with 3% H2O2 and blocked with 5% goat serum. Primary antibodies were incubated overnight at 4°C, followed by incubation with goat anti-rabbit IgG-HRP conjugate (Biocare Medical). Sections were then counterstained with hematoxylin. Antibodies and their dilutions are listed in Supplementary Table S2. All images were captured utilizing the Multiphoton Microscope (#TCS SP8, Leica, Wetzlar, Germany).

**Cell lines**

DLD1 (RRID: CVCL_0248), SW480 (RRID: CVCL_0546), HCT116 (RRID: CVCL_0291), LOVO (RRID: CVCL_0399), Caco-2 (RRID: CVCL_0025), SW1116 (RRID: CVCL_0544), and HT-29 (RRID: CVCL_0320) cells were obtained from the ATCC. All experiments were conducted using cells that had undergone 10 passages post-thawing. The cells were cultured in DMEM (Gibco, 11965118) supplemented with 10% (v/v) fetal bovine serum (Gibco, 16140071) and antibiotic-antimycotic (Gibco, 15240112) at 37 °C in a 5% $CO_2$ environment. All cell lines tested negative for mycoplasma and were authenticated through short tandem repeat (STR) genotyping.

**Lentivirus production**

Wildtype and mutant ADAT2 (A72S, E73V) plasmids were constructed by cloning the full-length coding DNA sequence (CDS) of the human *ADAT2* gene (NM_182503) into pLVX-Puro vector. ADAT2 sgRNAs were cloned into lentiCRISPR v2 vector. Wildtype HDAC7 was cloned into pLV-EF1a-IRES-Blast, and HDAC7 sgRNA was cloned into lentiCRISPR v2-Blast vector. Sequences of the sgRNAs are provided in Supplementary Table S3. For lentivirus production, HEK293T cells were used for lentivirus production. For HEK293T cells in a 10-cm dish, 6 µg of plasmid DNA, 4.5 µg of psPAX2 (Addgene no.12260), 1.5 µg of pMD2.G (Addgene no. 12259), and 20 µl of FuGENE ® HD (Promega) were mixed. The supernatant was collected at 48 after transfection.

**Cell viability and colony formation assay**

For cell viability assays, the 3-(4,5-dimethylthiazol-2-yl)-2,5-diphenyltetrazolium bromide

(MTT, 5 mg/mL; Invitrogen) assay was employed, and cells ($1 \times 10^3$per well) were seeded in a 96-well plate. After a 4-hour incubation with MTT, dimethyl sulfoxide (DMSO) was added, and absorbance was measured at 570 nm to determine cell viability. All experiments were conducted with 5 to 10 replicates per group. For colony formation assay, cells (300-500 per well) were plated onto a six-well plate. The culture medium was replaced every 3 days. After 10 to 14 days, colonies were fixed using ice-cold methanol and stained with 0.5% crystal violet. Cell colonies were analyzed using ImageJ (RRID: SCR_003070). All experiments were performed in triplicates.

**Transwell migration and invasion assays**

Transwell migration and invasion assays were conducted using uncoated and Matrigel-coated Transwell chambers (8.0 μm pore size; Corning, 354480), respectively. A total of $3 \times 10^5$ cells were suspended in 100 µL of serum-free medium and seeded into the upper compartment. The lower chamber was filled with 600 µL of complete culture medium containing 20% FBS. After approximately 48 hours for migration and 72 hours for invasion, the cells were washed with PBS, fixed in 4% paraformaldehyde for 15 minutes, and stained with crystal violet for 30 minutes. Three random fields were selected to count the stained cells for statistical analysis.

**Wound healing assay**

Sufficient cells were seeded into six-well plates to reach almost 100% confluency the following day. An artificial wound was generated in each well by scraping with a 200 µL sterile tip. Cells were then washed twice with PBS and incubated in FBS-free medium. Images of the scratches were captured at 0, 24, 48, and 72 hours. Wound closure (%) was

assessed using TScratch software.

**Apoptosis and cell cycle assay**

For cell apoptosis analysis, $5 \times 10^5$ cells were cultured in a 6-well plate and harvested after 48 hours. Apoptosis was assessed using the FITC Annexin V Apoptosis Detection Kit (BD Biosciences) following the manufacturer's instructions. For cell cycle assay, cells were serum-starved overnight and then stimulated with complete medium for 4 to 8 hours. The cells were collected, fixed in 70% ethanol at −20°C overnight, and stained with propidium iodide/RNase (50 µg/mL; BD Biosciences) for 15 minutes at room temperature in the dark. Flow cytometry was performed in FACSCelesta (BD Biosciences) and analyzed by FlowJo (version 10.8.1) for apoptosis and ModFit (version 5.0) for cell cycle.

**Organoid culture and ADAT2 overexpression/knockout**

Human colorectal cancer organoids were kindly provided by the Princess Margaret Cancer Center at the University of Toronto. The organoids ($5\times10^4$ per 20µL) were embedded in Matrigel (Corning) and cultured in a DMEM/F-12 (Gibco) medium supplemented with penicillin/streptomycin (100 U/mL), HEPES (10 mmol/L), Glutamax (Gibco), N2 supplement, B27 supplement, and conditioned medium containing WNT3A (50% v/v), R-spondin (10% v/v), and Noggin (100 ng/mL). Additional supplements included EGF (50 ng/mL), FGF10 (1.25 mmol/L), gastrin (10 nmol/L), N-acetylcysteine (1.25 mmol/L), nicotinamide (10 mmol/L), A-83-01 (500 nmol/L; Tocris Bioscience), and SB202190 (1 µmol/L). To manipulate ADAT2 expression, colorectal cancer organoids were transduced with ADAT2 lentivirus. The growth of the organoids was monitored using an inverted light microscope, and the surface area of the organoids in each random field was analyzed using

ImageJ.

**TUNEL staining**

TUNEL assay was conducted in accordance with the manufacturer's instructions (DeadEnd Fluorometric TUNEL, Promega, Madison, WI). Briefly, tumor sections were deparaffinized, rehydrated, and fixed in 4% paraformaldehyde for 15 minutes. 100 µL of proteinase K (20 mg/mL) was added to each slide incubated at room temperature for 30 minutes. Upon PBS wash, 100 µL of equilibration buffer was added to each slide at room temperature for 10 minutes. Slides were then incubated at 37°C for 60 minutes after adding 100 µL of TdT reaction mix and being covered by plastic coverslips. Reaction was stopped by 2X SSC, and slides were blocked by 0.3% hydrogen peroxide for 5 minutes. 100 µL of streptavidin-HRP (1:500 in PBS) was then added to each slide, incubated at room temperature for 30 minutes, followed by 100 µL of DAB solution for signal development.

**Clustered Regularly Interspaced Short Palindromic Repeats (CRISPR) domain–targeting assay**

All sgRNAs in this study were designed by Massachusetts Institute of Technology CRISPR Design (http://crispr.mit.edu). To minimize off-target effects, only sgRNAs with a quality score above 70 were used in this study. Sequences of the sgRNAs targeting constitutive coding exons of ADAT2 are provided in Supplementary Table 4. HCT116 or LOVO cells were infected with LentiV_Cas9_puro (Addgene no. 108100), followed by puromycin selection to derive cells stably expressing Cas9. Cas9-expressing human cell lines were infected with sgRNA linked with GFP (LRG2.1 vector, Addgene no. 108098). The percentage of GFP-positive cells was measured every 4 days from day 2 to day 14 after

infection by using the BD FACSCelesta flow cytometer (BD Biosciences). Final GFP and initial GFP percentages were used to calculate fold depletion. Two independent experiments were performed for each screening.

**Polysome profiling analysis**

Cells were exposed to 100 μg/mL cycloheximide (Sigma) for a duration of 15 minutes at 37°C, subsequently lysed on ice. The lysates underwent centrifugation at 13,000 g for 10 minutes at 4°C. A sucrose gradient ranging from 10% to 50% (w/v) was prepared, and gradient centrifugation was conducted in SW41 ultracentrifuge tubes (Beckman) for a period of 3 hours at 38,000 g and 4°C within an SW41 rotor. The gradients were fractionated with concurrent monitoring of absorbance at 254 nm (Bio-Rad). Fractions representing non-ribosomal particles (<40S), 40S, 60S, 80S, and polysomes were collected, and total RNA was isolated from each fraction utilizing Trizol reagent for subsequent analysis.

**RNA sequencing**

Total RNA was extracted and subsequently treated with DNase I, followed by quality assessments. The sequencing library was prepared using 2.0 μg of total RNA from each sample at Aksomics (Shanghai, China). Briefly, mRNA enrichment was achieved employing the NEBNext® Poly(A) mRNA Magnetic Isolation Module kit. The library derived from the enriched RNA was constructed utilizing the KAPA Stranded RNA-Seq Library Prep Kit (Illumina) and subsequently evaluated with the Agilent 2100 Bioanalyzer. The final quantification of the library was performed via qPCR. After denaturation with 0.1 M NaOH, 8 pM of single-stranded DNA was amplified in situ using the NovaSeq S4 Reagent Kit. The resulting fragments were sequenced on the Illumina NovaSeq 6000

platform for 150 cycles. Post-sequencing, the results were compared to the known transcriptome using StringTie, and transcript abundances at gene and transcript levels were quantified employing Ballgown. Expression levels were expressed as FPKM (Fragments Per Kilobase of transcript per Million mapped fragments).

**Ribosome-sequencing (Ribo-seq)**

Cells were treated with 100 μg/mL cycloheximide for 5 minutes at 37°C, and unprotected mRNA regions within cells were digested with RNase I. Ribo-seq analysis was conducted by Omicsmart (China). Intact mRNA-ribosome complexes were sequenced using Illumina HiSeq 4000. Reads aligning to human rRNAs, snoRNAs, snRNAs, and tRNAs were filtered out, and the remaining reads were mapped to the human genome via Bowtie2 (v2.3.4.3). FeatureCounts (v1.6.4), with parameters M–fracOverlap 0.4–largestOverlap, was employed to quantify the expression levels of protein-coding genes. Gene Set Enrichment Analysis (GSEA) was performed using the GSEA software, with the KEGG MEDICUS pathway database as the reference. Genes were ranked using the Signal-to-Noise ratio method. To estimate translation efficiency (TE), RNA-seq was conducted on total RNA samples. The Integrative Genomics Viewer (IGV) was used to visualize TE as the enrichment ratio of Ribo-seq to RNA-seq.

**tRNA-sequencing**

tRNA-seq was conducted on the Illumina NextSeq 500 platform by Aksomics (Shanghai, China). Over 2 μg of total RNA was separated using 7.5% urea-PAGE and tRNA fragments ranging from 60 to 100 nucleotides were isolated. To ensure the accurate detection of A-to-I editing (which is read as A-to-G changes in sequencing data), it is critical to overcome

reverse transcription stops and misincorporation caused by abundant tRNA modifications. Due to post-transcriptional modifications that hinder cDNA synthesis [1], an RNA pre-treatment kit (Arraystar) was employed to demethylate tRNA $m^1A$, $m^1G$, and $m^3C$ residues, thus facilitating efficient cDNA reverse transcription. This demethylation step is essential to reduce reverse transcription artifacts and allow for the unambiguous mapping of reads containing inosine-derived guanosine (I-G). Demethylated tRNA was partially hydrolyzed in accordance with the Hydro-tRNA-seq protocol and subsequently re-phosphorylated [2]. tRNA was partially hydrolyzed using $Na_2CO_3/NaHCO_3$ buffer, which generates fragments of optimal length for sequencing and minimizes issues related to translating through tRNA modifications, followed by dephosphorylation with calf intestinal alkaline phosphatase (CIP), and re-phosphorylation with T4 polynucleotide kinase. Purified tRNA fragments were then utilized to construct barcoded small RNA libraries using the NEBNext Small RNA Library Prep Set for Illumina (New England Biolabs). These libraries were validated and quantified with an Agilent 2100 Bioanalyzer, pooled in equal proportions, and sequenced on the Illumina NextSeq 500 system. For data analysis, reads were aligned to the reference genome permitting for a limited number of mismatches, enabling the specific identification of A-to-G mismatches at known ADAT2 target sites (corresponding to A-to-I modification).

**Ribosome-Nascent Chain Complex qPCR (RNC-qPCR)**

Cells were pre-treated with cycloheximide at a concentration of 100 µg/ml for 15 minutes at 37°C, followed by lysis in lysis buffer on ice for 30 minutes. The lysis buffer was prepared by adding 1% Triton X-100 to ribosome buffer, which included 20 mM HEPES-KOH (pH 7.4), 15 mM $MgCl_2$, 200 mM KCl, 100 µg/ml cycloheximide, and 2 mM

dithiothreitol. The cell lysate was subsequently extracted by centrifugation at 4°C for 5 hours at 190,000 g. The cell pellets were then harvested and subjected to extraction using TRIzol reagent. Total RNA, isolated from both the input control and RNC samples, was utilized for RT-qPCR for subsequent analysis.

**Luciferase reporter assay**

The wild-type HDAC7 full-length CDS were cloned into the pcDNA3.1 vector. Mutant HDAC7 constructs, with specific nucleotide substitutions (ACC to ACG, GCC to GCG, CCC to CCG, TCC to TCG, CTC to CTG, ATC to ATA, GTC to GTG, and CGC to CGG), were inserted into the plenti-EF1a-FH-CBH vector. Cells were seeded in 24-well plates at a density of $1 \times 10^5$ cells per well. The transcriptional activity of β-catenin within the cells was assessed using TOPflash/FOPflash and LEF-1 luciferase reporter assays, which were co-transfected with Renilla luciferase via FuGENE® 4K. All Firefly luciferase readings were normalized to Renilla luciferase activity. The FOPflash reporter served as a negative control. The Dual-Luciferase Reporter Assay System (Bio-Rad, Hercules, CA) was employed to measure the activities of Firefly and Renilla luciferases after 24 hours post-transfection.

**Construction and validation of codon-mutated reporters**

Mutant HDAC7 luciferase reporter was generated by site-directed mutagenesis to replace all adenosine-ending codons within the HDAC7 coding sequence that are decoded by A-to-I modified tRNA species (ACC, GCC, CCC, TCC, CTC, ATC, GTC, CGC) with their synonymous counterparts (ACG, GCG, CCG, TCG, CTG, ATA, GTG, CGG), preserving the exact amino acid sequence. To control for the effects on mRNA expression, the steady-

state mRNA levels of luciferase gene from wildtype and mutant reporters were quantified by RT-qPCR.

### Co-immunoprecipitation and mass spectrometry

Cells were lysed using RIPA buffer. For co-immunoprecipitation, 1 mg of tissue lysates was incubated overnight at 4°C with 1 μg of antibodies against HDAC7, ADAT2, Flag, or normal IgG. To perform the pulldown assay, the mixture was incubated with 50 μL of Protein A/G Mix Magnetic Beads (Merck Millipore, Burlington) for 1 hour at 4°C. Following incubation, the beads were washed with RIPA buffer, and proteins were eluted by heating in SDS-PAGE loading buffer. The eluted proteins were separated via SDS-PAGE and subjected to silver staining (Thermo Fisher Scientific, 24612). For mass spectrometry analysis, the silver-stained bands were excised, digested with trypsin, and analyzed using nano-Ultra Performance Liquid Chromatography (EASY-LC 1200) coupled to a Q Exactive HFX Orbitrap mass spectrometer (Thermo Fisher Scientific). Raw mass spectrometry data files were processed with Proteome Discoverer software (version 2.4.0.305) utilizing the integrated Sequest HT search engine against the UniProt FASTA database. Up to two missed cleavages were permitted. The false discovery rate was set at 0.01, with all other parameters set to default. The enrichment of Gene Ontology biological process terms among the protein candidates was evaluated using the topGO package in R. Additionally, GSEA was performed employing GSEA software, referencing the KEGG MEDICUS pathway database.

### Puromycin incorporation assay

Cells were seeded in 6-well plates at a density of $3 \times 10^5$ cells per well. At the endpoint,

cells were incubated with puromycin at a concentration of 1 μg/ml for a duration of 30 to 60 minutes, followed by lysis in RIPA buffer. The lysate was subjected to separation via SDS-PAGE, transferred onto a PVDF membrane, and analyzed using an anti-puromycin antibody. A loading control gel was prepared concurrently and stained with Coomassie Blue solution.

**Vesicle-like PLGA-based nanoparticle (VNP) formulation and treatment**

VNPsiRNA was prepared using a double emulsion technique [3]. Typically, an aqueous solution of siRNA (15 nmol, 25 μL) was emulsified through sonication over an ice bath for 1.0 minute in 0.5 mL of chloroform containing 2.0 mg of DOTAP and 25.0 mg of the amphiphilic polymer mPEG-b-PLGA. This primary emulsion was subsequently emulsified in 5 mL of water via sonication (65 W for 2 minutes) over an ice bath. Following this, the chloroform was removed using a rotary evaporator. The resulting nanoparticle dispersion (designated as VNPsiRNA) was transferred to an ultrafiltration device (Amicon® Ultra-15, MWCO 100 K) and centrifuged to eliminate any free compounds. After washing with water three times (each 5 mL), the final VNPsiRNA was stored at 4°C.

**Mouse colonoscopy**

For CRC mouse models, colonoscopy was performed using a KARL STORZ ENDOSCOPE (length: 10 cm, diameter: 19 mm). Mice were deprived of food for a minimum of 8 hours on the day preceding the procedure. Anesthesia was induced via inhalation of 4% isoflurane through a Gas Filter Canister. The endoscope was carefully inserted through the mouse's anus, utilizing $CO_2$ insufflation (pressure maintained below 10-15 mmHg, gas flow between 5-10 L/min) to ensure colon inflation. The colon was

rinsed with warm PBS as needed during the advancement of the endoscope to the point of the colon's curvature. The endoscope was then gradually withdrawn while images were captured until the anus was reached. All procedures received approval from the Animal Experimentation Ethics Committee of The Chinese University of Hong Kong (Approval numbers: 23-138-MIS and 24-304-GRF).

## Mouse Models

### *Subcutaneous xenograft models*

Mice were randomly assigned to groups and maintained under specific pathogen-free conditions at 22 °C, with a humidity range of 40–70%, following a 12-hour light–dark cycle, and having unrestricted access to chow and water. For subcutaneous xenografts, CRC cells transduced with lentiviral-carrying sgCTL and sgADAT2, or vector and ADAT2-OE, were injected subcutaneously into the left and right dorsal flanks of 4- to 6-week-old male NU/J mice (RRID: IMSR_JAX:002019) ($3 \times 10^6$ cells in 0.1 mL phosphate-buffered saline per mouse). Tumor size was measured every two days using a digital caliper. Tumor volume ($mm^3$) was calculated using the formula: $V=0.5 \times L \times W^2$, where L represents the longest diameter and W represents the shortest diameter. At the endpoint, tumors were harvested and weighed. All animal experiments received approval from the Animal Experimentation Ethics Committee of The Chinese University of Hong Kong (Ref. No. 23-138-MIS and 24-304-GRF).

### *Transgenic ADAT2 knockout mice*

The whole body *ADAT2*$^{KO}$ (*ADAT2*$^{+/-}$) mice were generated by CRISPR/Cas9 by GemPharmatech Co., Ltd (Nanjing, China). Conditional ADAT2 transgenic mice were

crossed to *Villin-Cre* mice to generate colon-specific ADAT2 transgenic mice. Validation was performed by RT-PCR. To induce CRC, mice at the age of 7 ~ 8-week-old were injected intraperitoneally with azoxymethane (AOM) (10 mg/kg body weight). Five days later, mice were given 1.5% dextran sulfate sodium (DSS) (MP Biomedicals) in drinking water for 5 days followed by 2 weeks of regular drinking water. DSS treatments were repeated twice. For the chemotherapy experiment, 5-FU (50 mg/kg) and OXA (3.25 mg/kg) twice a week were administered following the final round of DSS treatment. After 4 weeks of chemotherapy, the mice were anesthetized and subsequently sacrificed.

For $Apc^{Min/+}$ model, $ADAT2^{+/-}$mice were bred to C57BL/6J $Apc^{Min/+}$ mice (RRID: IMSR_JAX:002020) to generate $Apc^{Min/+}ADAT2^{KO}$ mice. At harvest, tumor number was counted and tumor dimensions were measured with a digital caliper. Tumor volume ($mm^3$) was calculated using the following formula: d2 × D/2, where d and D are the shortest and longest diameter in mm, respectively. The tumor burden was calculated as the total tumor volume in each individual mouse. Colon and tumor tissues were stored in -80°C or fixed in 10% formalin for downstream analysis. Histology was scored after H&E staining by a pathologist blinded to the nature of samples. All animal studies were performed in accordance with the guidelines approved by the Animal Experimentation Ethics Committee of The Chinese University of Hong Kong (Ref. No. 23-138-MIS and 24-304-GRF).

***Syngeneic animal model***

In the syngeneic animal model, eight days following cell injection, the mice were administered treatments with chemotherapeutic agents, including 5-FU (50 mg/kg) and OXA (7.5 mg/kg), which were given by intraperitoneal injection twice a week.

Concurrently, the mice received treatments of VNP-siNC or VNP-siADAT2 every four days (on days 7, 11, 15, 19, 23, and 27) via intratumoral injection at a dose of 3 OD per mouse. Vesicle-like PLGA-based nanoparticles (VNP) were assembled by Guangzhou Kelan Biotechnology Co., Ltd. (Guangzhou, China). The siRNA sequences were obtained from GenePharma Co., Ltd. (Shanghai, China). The sequences of human ADAT2 siRNAs were as follows: sense strand: GCUGUCUUAUGGUCUACAATT; antisense strand: UUGUAGACCAUAAGACAGCTT. VNP-siRNA treatments were administered into each mouse using a 27G needle. All experimental procedures were approved by the Animal Experimentation Ethics Committee of The Chinese University of Hong Kong (Ref. No. 24-304-GRF).

**Statistical analysis**

All statistical analyses were conducted using GraphPad Prism 9.5. Data were presented as means ± standard deviation (SD). Statistical comparisons between two groups were performed using unpaired Student T-test, Mann-Whitney U test, or Wilcoxon's rank sum test. Statistical comparisons between more than two groups were performed with One-way analysis of variance (ANOVA) with Fisher's LSD test. Statistical comparisons between two curves were compared by Two-way analysis of variance test. A multivariable Cox proportional hazards regression model was used to assess the independent prognostic value of ADAT2 expression and adjusted for clinicopathological variables, including age, gender, tumor size (for Cohort IV), and TNM stage. The cases with missing values for any of the variables (ADAT2 expression, age, gender, tumor size (for Cohort IV) or TNM stage) were excluded. The number of complete cases analyzed in Cohort I (TCGA) was N=274 and for Cohort IV (TMA) was N=157. Histological assessments were analyzed by Chi-square test.

P values <0.05 were considered to be statistically significant.

**References:**


1 Zheng G, Qin Y, Clark WC, Dai Q, Yi C, He C, *et al.* Efficient and quantitative high-throughput tRNA sequencing. Nat Methods 2015;**12**:835-7.

2 Gogakos T, Brown M, Garzia A, Meyer C, Hafner M, Tuschl T. Characterizing Expression and Processing of Precursor and Mature Human tRNAs by Hydro-tRNAseq and PAR-CLIP. Cell Rep 2017;**20**:1463-75.

3 Zhang W, Zhang Y, Luo Y, Chen S, Huang Q, Cao Z, *et al.* A nanoconfined loading strategy for highly efficient siRNA delivery and cancer therapy. Nano Today 2022;**43**:101418.
